# Mast cells help organize the Peyer’s patch niche for induction of IgA resonses

**DOI:** 10.1126/sciimmunol.adj7363

**Published:** 2024-03-01

**Authors:** Marco De Giovanni, Vivasvan S. Vykunta, Adi Biram, Kevin Y. Chen, Hanna Taglinao, Jinping An, Dean Sheppard, Helena Paidassi, Jason G. Cyster

**Affiliations:** 1 Howard Hughes Medical Institute and Department of Microbiology and Immunology, University of California, San Francisco, San Francisco, CA 94143, USA.; 2 Division of Immunology, Transplantation and Infectious Diseases, IRCCS Ospedale San Raffaele, Milan, Italy.; 3 Medical Scientist Training Program, School of Medicine, University of California, San Francisco, San Francisco, CA 94143, USA.; 4 Lung Biology Center, Department of Medicine, University of California San Francisco, 1550 4^th^ Street, San Francisco, CA 94158, USA.; 5 CIRI, Centre International de Recherche en Infectiologie, Univ Lyon, Inserm, U1111, Université Claude Bernard Lyon 1, CNRS, UMR5308, ENS de Lyon, France.

## Abstract

Peyer’s patches (PPs) are lymphoid structures situated adjacent to the intestinal epithelium, which support B cell responses that give rise to many intestinal IgA secreting cells. Induction of isotype switching to IgA in PPs requires interactions between B cells and TGFβ-activating conventional dendritic cells type 2 (cDC2s) in the subepithelial dome (SED). However, the mechanisms promoting cDC2 positioning in the SED are unclear. Here we found that PP cDC2s express GPR35, a receptor that promotes cell migration in response to various metabolites including 5-hydroxyindoleacetic acid (5-HIAA). In mice lacking GPR35, fewer cDC2s were found in the SED, and frequencies of IgA^+^ germinal center (GC) B cells were reduced. IgA plasma cells were reduced in both the PPs and lamina propria. These phenotypes were also observed in chimeric mice that lacked GPR35 selectively in cDCs. GPR35-deficiency led to reduced coating of commensal bacteria with IgA, and reduced IgA responses to cholera toxin. Mast cells were present in the SED and mast cell-deficient mice had reduced PP cDC2s and IgA^+^ cells. Ablation of tryptophan hydroxylase 1 (Tph1) in mast cells to prevent their production of 5-HIAA similarly led to reduced PP cDC2s and IgA responses. Thus, mast cell-guided positioning of GPR35^+^ cDC2s in the PP SED supports induction of intestinal IgA responses.

## One sentence summary

Intestinal IgA production depends on GPR35-expressing cDC2 positioning guided by mast cells within Peyer’s patches

## Introduction

IgA is the major antibody isotype produced at mucosal surfaces where it is transcytosed by epithelial cells to protect from luminal pathogens. In the intestine, commensal-binding IgA has a strong influence on the diversity and function of the microbiome (1, 2). Many IgA producing plasma cells are generated in Peyer’s patches (PPs), lymphoid tissues of the small intestine of which there are approximately 100 in humans, and 5–10 in mice (3). The epithelium overlying PPs contains specialized ‘microfold’ epithelial cells (M cells) that transcytose gut antigens to a compartment rich in myeloid cells and B cells called the subepithelial dome (SED) (3). Beneath the SED are the B cell follicles and their associated germinal centers (GCs). PPs promote B cell isotype switching to IgA and many PP GC B cells are IgA^+^. Induction of IgA class switch recombination is strongly dependent on TGFβ1 (3). Activation of latent TGFβ1 requires binding by αv-containing integrins (4). Previous work established an important role for αvβ8 integrin expressing conventional dendritic cells type 2 (cDC2) in the SED in promoting TGFβ1 signaling in PP B cells (5). More recent work identified additional myeloid cell types within the SED, in particular lysozyme (Lyz) expressing cells of monocyte origin termed Lyz DC and Lyz macrophages (Mac) (6, 7). The role of these additional myeloid cells in the IgA response is unclear.

B cell recruitment to the SED is dependent on the chemokine receptor CCR6 (5). The CCR6 ligand, CCL20, is constitutively expressed by the follicle associated epithelium (FAE) (8, 9). In mice lacking CCR6 in B cells, there are fewer B cells in the SED, reduced interactions with αvβ8^+^ SED cDC2s, and reduced IgA responses (5). However, CCR6-deficieny was not found to have a reproducible effect on cDC positioning in the SED making it unclear how cDC2s localize in this niche (10).

The G-protein coupled receptor GPR35 was recently shown to contribute to the recruitment of neutrophils to sites of inflammation (11). GPR35 binds a variety of small molecules including tryptophan metabolites although most of the identified GPR35 ligands have low potency and their role *in vivo* is unclear (12). In recent work, we identified the serotonin metabolite 5-hydroxyindoleacetic acid (5-HIAA) as a nanomolar potent ligand for GPR35 (11, 13). Serotonin is generated from tryptophan by the action of tryptophan hydroxylases (Tph1 and Tph2) and it is metabolized to 5-HIAA by monoamine oxidases types a and b (Maoa and Maob). A key cell type that expresses Tph1 and Maob and that can release 5-HIAA at sites of inflammation is the mast cell (13). Mast cells have been identified in PPs, but their function has been unclear (14, 15).

Here we investigated the mechanisms underlying cDC2 positioning in the PP SED. We found that GPR35 is highly expressed by PP cDC2s, and these cells respond *in vitro* to 5-HIAA. Deficiency in GPR35 globally or within DC led to a reduction in SED cDC2s, GC IgA^+^ cell frequencies, lamina propria IgA plasma cells, and intestinal IgA. Mast cells were identified within PP SEDs and loss of Tph1 from mast cells led to a defect in the intestinal IgA response. These findings establish a role for GPR35 as a chemoattractant receptor in cDC2s and for both GPR35 and mast cells as organizers of the PP SED to support intestinal IgA responses.

## Results

### PP cDC2 express GPR35 and depend on the receptor for homeostasis

**Figure 1.**
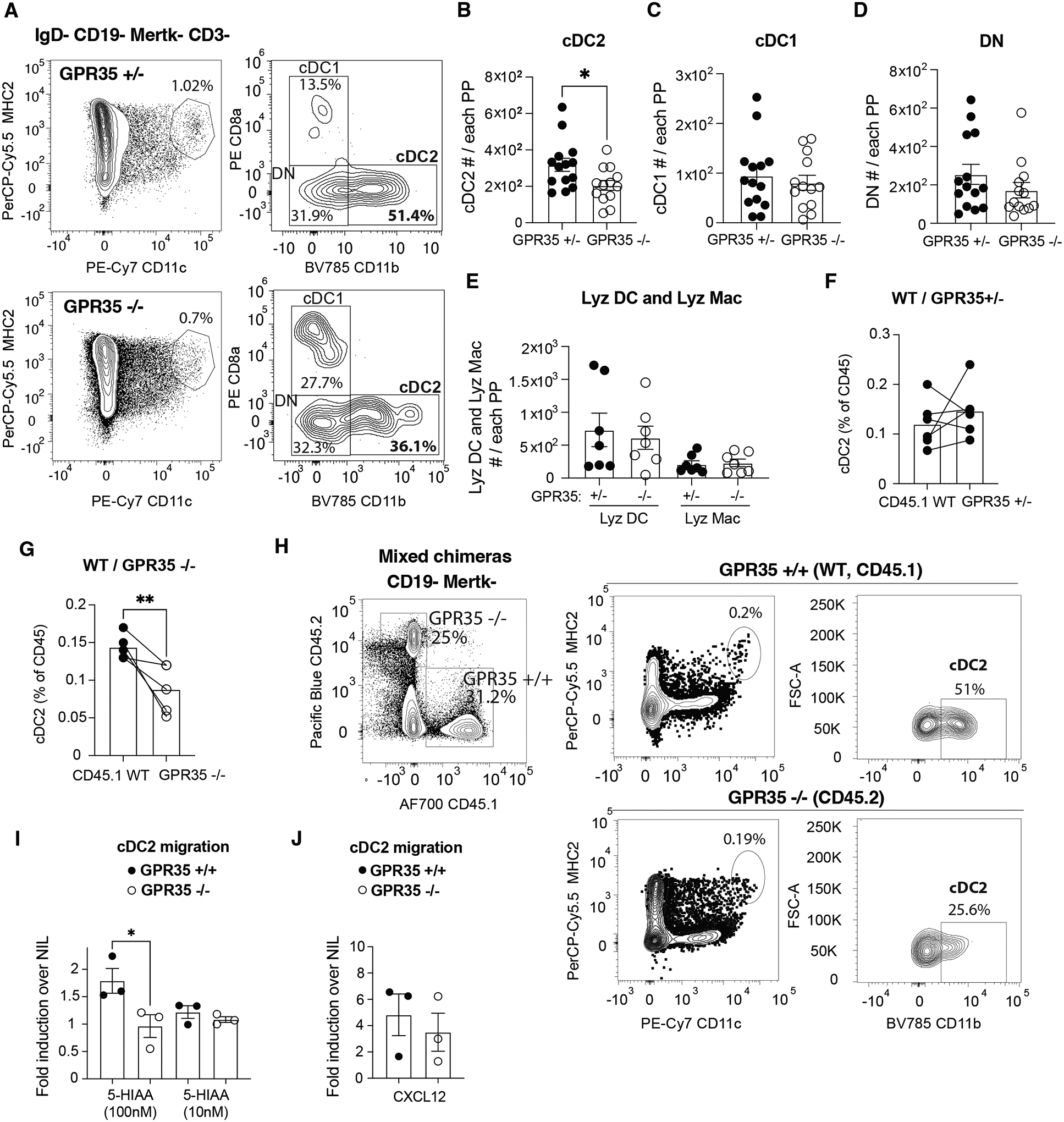
GPR35 promotes cDC2 maintenance in Peyer patches. **A**. Flow cytometry plots showing cDC percentages in PPs from control (GPR35^+/−^, top) and GPR35^−/−^ (bottom) mice. **B-E**. Absolute numbers of **(B**) cDC2 (IgD^−^ CD19^−^ CD3^−^ Mertk^−^ CD11c^+^ MHC2^+^ CD11b^+^), (**C**) cDC1 (IgD^−^ CD19^−^ CD3^−^ Mertk^−^ CD11c^+^ MHC2^+^ CD8a^+^), (**D**) DN DC (IgD^−^ CD19CD3^−^ Mertk^−^ CD11c^+^ MHC2^+^ CD11b^−^ CD8a^−^), (**E**) Lyz DC (IgD^−^ CD19^−^ CD3^−^ BST2^+^ CD11c^+^ MHC2^+^ CD4^−^) and Lyz Macrophages (Mac, IgD^−^ CD19^−^ CD3^−^ BST2^+^ CD11c^+^ MHC2^+^ CD4^+^) in PPs from GPR35^+/−^ and GPR35^−/−^ mice. *n*= 14 GPR35^+/−^ in **B-D**; *n*=13 GPR35^−/−^ in **B-D**; *n*=7 in **E**. Data are pooled from 3 independent experiments. **F-H**. Quantification of cDC2 % (out of cDC) in (**F**) CD45.1 WT/ CD45.2 GPR35^+/−^ and (**G**) CD45.1 WT/CD45.2 GPR35^−/−^ mixed BM chimeras and (**H**) example flow cytometry plots. *n*=5 in **F**; *n*=4 in **G**. Data are pooled from 2 independent experiments. **I, J**. Quantification of GPR35^+/+^ and GPR35^−/−^ cDC2 migration to (**I**) 5-HIAA and (**J**) CXCL12 at the indicated concentrations in Transwell migration assays. Each dot represents one independent experiment. Data are pooled from 3 independent experiments and are plotted as fold migration over nil to allow comparisons between experiments that have different baseline migration. * p<0.05; ** p<0.005; *** p<0.0005. Data are presented as mean ± SEM. Two-tailed unpaired t-tests (**B-E**), paired- t-tests (**F, G**), and two-way ANOVA with multiple comparisons test (I) was performed.

To gain insights into guidance receptors that may be involved in cDC2 function within PPs, we interrogated a published PP scRNAseq dataset (16). This analysis showed high expression of *Gpr35* in cDC2 but not cDC1 (Supplementary. Fig. 1A). Flow cytometric analysis using an antibody raised against the GPR35 C-terminal cytoplasmic domain (11) confirmed high GPR35 expression by PP cDC2s compared to other cDCs (Supplementary. Fig. 1B). Analysis of PPs from GPR35 knockout (KO) mice revealed a reduction in the number of CD11b^+^ cDC2s per PP (Fig. 1A, B and Supplementary Fig.1C). PP double negative (DN) cDC and CD8^+^ cDC1 numbers were not significantly affected (Fig. 1C, D and Supplementary. Fig. 1D, E). GPR35-deficient mice had normal numbers of PPs and intact total cell numbers per PP (Supplementary Fig. 1F, G). *Gpr35* expression was also detected in Lyz DC and Lyz Mac (Supplementary. Fig. 1A). However, numbers of PP Lyz DC, identified as MHC2^+^CD11c^+^BST2^+^CD4^−^ and PP Lyz Mac, identified as MHC2^+^CD11c^+^BST2^+^CD4^+^, were unaltered (Fig. 1E). Altered dependence on GPR35 may be a consequence of strong differences in expression of other chemoattractant receptors (Supplementary Fig. 1A). In BM chimeras made using equal mixtures of CD45.1 (WT) and GPR35 HET or GPR35 KO BM, there was a selective deficit in GPR35 KO cDC2 representation (Fig. 1F, G). Moreover, cDC2 frequencies and numbers in spleen were not affected by GPR35-deficiency (Supplementary Fig. 1H, I). Thus, GPR35 is required cell-intrinsically for cDC2 homeostasis in PPs.

### PP cDC2s require GPR35 for positioning in the SED

**Figure 2.**
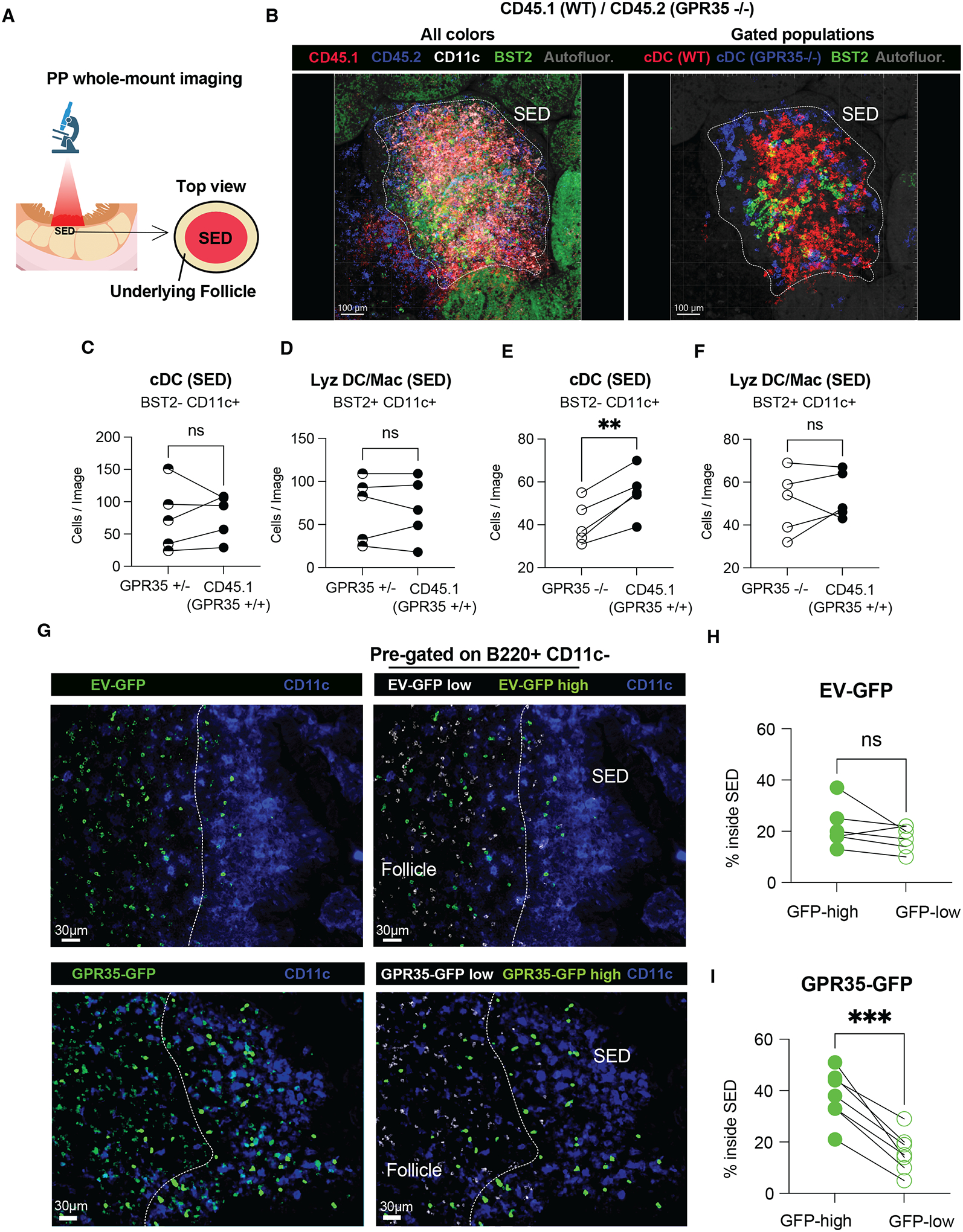
GPR35 drives cDC localization within SED. **A**, **B**. Schematic representation (**A**) and confocal maximum intensity projections (**B**) of whole-mount PP SED from CD45.1 WT/CD45.2 GPR35^−/−^ mixed chimera. Images show CD45.2 (blue), CD45.1 (red), BST2 (green), and CD11c (white) signal (**B**, left); or cDC WT (red), cDC GPR35^−/^(blue) and BST2^+^ Lyz DC/Mac (green) gated populations (**B**, right). The white dashed line delimits the SED and was depicted based on CD11c positivity. **C-F.** Quantification of BST2^−^ CD11c^+^ (cDCs, **C** and **E**), BST2^+^ CD11c^+^ (Lyz DC/Mac, **D** and **F**) cells from images of the type shown in **B** and Supplementary Movies 1 and 2. *n*=5. Data are pooled from 2 independent experiments. SED=subepithelial dome. * p<0.05; ** p<0.005; *** p<0.0005. Data are presented as mean ± SEM. **G**. Immunofluorescence micrographs showing PPs from EV-GFP (top) and GPR35-GFP (bottom) overexpressing chimeric mice. GFP total (green, left panels) and CD11c (purple, all panels) staining are shown. Masked channels of GFP low (white, right panel), GFP high (green, right panel) are also shown. Dashed line shows boundary between SED and follicle. **H, I**. Quantification of EV-GFP (**H**) and GPR35-GFP (**I**) high and low cell % inside the SED from images of the type shown in A. *n*=6 in **H**; *n*=7 in **I**. Data are pooled from 2 independent experiments. SED=subepithelial dome. **O**. Quantification of CT (cholera toxin)-binding IgA relative abundance in CT-immunized GPR35^+/−^, GPR35^−/−^ and non-immunized controls (no CT). *n*=10 (GPR35^+/−^); *n*=16 (GPR35^−/−^); *n*=3 (no CT). Data are pooled from 2 independent experiments and co-caged mice are shown in the same color. Data were pooled from 3 independent experiments. * p<0.05; ** p<0.005; *** p<0.0005. Data are presented as mean ± SEM. Paired-t-tests were used.

GPR35 can support cell migration in response to tryptophan metabolites including 5-HIAA (*11–13*). To test whether GPR35 sustains migration of cDC2s within PP, we performed transwell chemotaxis assays and found that PP cDC2s migrated to nanomolar gradients of 5-HIAA in a GPR35-dependent fashion (Fig. 1H, I and Supplementary. Fig. 1J, K). DCs in PPs are situated in both the SED and the interfollicular (T cell rich) region. By examining PP tissue sections from GPR35 KO mice, we could detect a reduction of CD11c^+^CD11b^+^BST2^−^ cDCs in the SED (Supplementary Fig. 1L, M). Littermate (GPR35^+/−^) mice were used as cage-matched controls. A selective reduction of CD11c^+^CD11b^+^ GPR35-deficient cells was observed within the SED of CD11c-GFP / GPR35 KO mixed chimeras suggesting GPR35 plays a cell-intrinsic role in cDC2 accumulation in the SED (Supplementary Fig. 1N, O). To further test if the cDC2 deficiency in the SED reflected a cell intrinsic role for GPR35, PPs from mixed CD45.1 WT:CD45.2 GPR35^+/−^ control or CD45.1 WT:CD45.2 GPR35^−/−^ BM chimeras were analyzed by whole mount imaging (Fig. 2A, B). Due to limitations in the number of colors that could be used in the whole mount analysis, we did not distinguish between cDC1 and cDC2. However, cDC1s are very rare in the SED compared to cDC2s (6). In this analysis, CD45.1 WT cDCs were detected in SEDs in greater abundance than GPR35 KO cDCs (Fig. 2B, C and E, Supplementary Movie 1–2). BST2^+^CD11c^+^ Lyz DC and Lyz Mac present in the same images were unaffected by GPR35-deficiency (Fig. 2D, F).

As another approach to test the impact of GPR35 on cell positioning in the SED we examined cell distribution in retroviral GPR35 over-expression BM chimeras. B cells are the most frequent cell type in PPs and since they do not normally express GPR35, we asked if GPR35 expression was sufficient to cause them to localize in the SED. We examined the distribution of GPR35 over-expressing (GFP reporter high) B cells compared to control (GFP low) B cells in the same PPs. This analysis revealed that GPR35 highly expressing B cells were over-represented in the SED (Fig. 2G–I). These data suggest that GPR35 may be both necessary and sufficient to promote cDC2 positioning in the SED.

### GPR35-deficiency is associated with a reduced PP and intestinal IgA response

**Figure 3.**
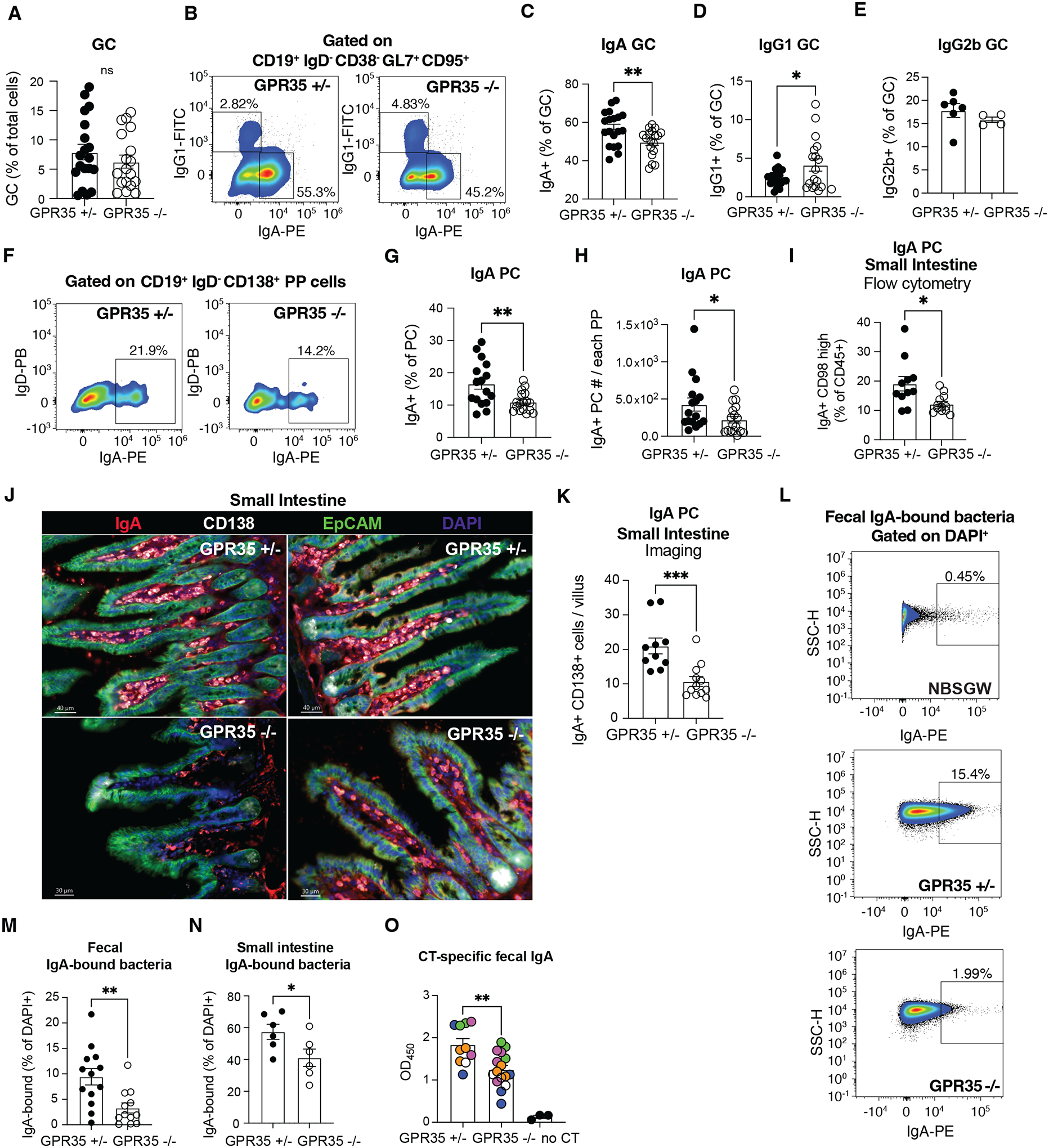
GPR35 supports intestinal IgA B cell responses. **A.** Quantification of GC cells % (CD19^+^ IgD^−^ CD38^−^ Gl7^+^ CD95^+^ out of total cells) in PPs from control (GPR35^+/−^) and GPR35^−/−^ mice using indicated markers. **B**. Flow cytometry plots showing % of IgA^+^ and IgG1^+^ GC B cells. **C-E**. Summary graphs showing % GC B cells that were IgA^+^ (**C**) IgG1^+^ (**D**) or IgG2b^+^ (**E**) in PPs from GPR35^+/−^ and GPR35^−/−^ mice. *n*=19 (GPR35^+/−^ **A**, **C**, **D**); *n*=18 (GPR35^−/−^, **A**, **C**, **D**); *n*=6 (GPR35^+/−^, **E**), *n*=4 (GPR35^−/−^, **E**). Data are pooled from 4 (**A**, **C**, **D**) or 2 (**E**) independent experiments. **F**. Representative flow cytometry plots showing % of IgA^+^ (out of CD19^+^ IgD^−^ CD138^+^ plasma cells). **G, H**. Quantification of IgA^+^ % (out of CD19^+^ IgD^−^ CD138^+^ plasma cells, **G**) and absolute numbers (**H**) of IgA^+^ plasma cells in PPs from GPR35^+/−^ and GPR35^−/−^ mice using gating as in F. *n*=16 (Gpr35^+/−^, **G, H**); *n*=17 (GPR35^−/−^, **G, H**). Data are pooled from 4 independent experiments. **I-K**. Quantification of IgA^+^ CD98 high plasma cell % (out of CD45^+^) by flow cytometry (**I**) and of IgA^+^ CD138^+^ plasma cells quantification by imaging (**J**, **K**) in the small intestine of GPR35^+/−^ and GPR35^−/−^ mice. (**J**) Shows two representative epifluorescence images of data quantified in (**K**) of small intestinal villi from GPR35^+/−^ (upper) and GPR35^−/−^ (lower) mice. IgA (red), CD138 (white), EpCAM (green) and DAPI (blue) staining are shown. *n*=10 (GPR35^+/−^, **I, K**); *n*=12 (GPR35^−/−^, **I, K**). **L**. Representative flow cytometry plots showing % (out of DAPI^+^) of IgA-bound fecal bacteria from lymphocyte-deficient NBSGW (negative control, top), GPR35^+/−^ (middle) or GPR35^−/−^ (bottom) mice. **N-M.** Quantification of IgA-bound fecal (**M**) or small intestine (**N**) bacteria in GPR35^+/−^ and GPR35^−/−^ mice. *n*=13 (GPR35^+/−^, **M**); *n*=12 (GPR35^−/−^, **M**); *n*=6 (GPR35^+/−^ and GPR35^−/−^, **N**). Data are pooled from 3 (**M**) or 2 (**N**) independent experiments. Data are representative of 3 independent experiments. * p<0.05; ** p<0.005; *** p<0.0005. Data are presented as mean ± SEM. Two-tailed unpaired t-tests were performed.

Given the important role of cDC2s in promoting IgA isotype switching in PPs we next examined the impact of GPR35-deficiency on the IgA response. Co-housed littermate control and GPR35 KO mice were used for these studies. GPR35 KO mice showed a reduction in the frequency of IgA^+^ GC B cells in PPs (Fig. 3A–C). This was accompanied by an increased frequency of IgG1^+^ GC B cells consistent with a defect in switching to IgA (Fig. 3D). IgG2b^+^ GC B cell frequencies were unaltered (Fig. 3E). Importantly, GPR35-deficient mice showed a 2-fold reduction in the frequency and number of CD138^+^ IgA^+^ plasma cells in PPs (Fig. 3F–H). A reduction in plasma cell numbers in the lamina propria was identified by both FACS analysis and immunofluorescence microscopy (Fig. 3I–K). Flow cytometric analysis revealed a significant reduction in IgA-coated fecal and small intestine bacteria in GPR35-deficient mice (Fig 3L–N). GPR35-deficient mice also showed reduced total fecal IgA (Supplementary Fig. 2A). Antigen-specific IgA responses to cholera toxin immunization are almost entirely T cell-dependent (17). GPR35-deficient mice mounted a reduced antigen-specific intestinal IgA response following oral cholera toxin challenge (Fig. 3O), consistent with GPR35 supporting de novo GC-dependent IgA responses. BM chimera experiments showed that GPR35-defiency in hematopoietic cells, but not radioresistant host cells, led to reduced IgA^+^ GC B cells, reduced IgA^+^ plasma cells in PPs and reduced IgA^+^ bacteria (Supplementary Fig. 2B–E). Furthermore, the number of cDC2s, IgA GC and IgA plasma cells were not affected in mesenteric LNs in the absence of GPR35 (Supplementary Fig. 2F–H). These findings are consistent with GPR35 in cDC2s being needed to support PP IgA^+^ B cells, plasma cells and intestinal IgA responses, and suggest that the reductions observed are not dependent on pre-existing microbiome alterations in GPR35-deficient hosts.

Several studies have revealed a critical role of αvβ8 integrin-mediated TGFβ activation by mucosal cDC (both cDC1 and cDC2) for the promotion of IgA responses in different contexts (5, 18–20). In PPs, αvβ8^+^ cDC2s have been most strongly implicated in induction of the IgA switch (5). Flow cytometry analysis for expression of an Itgb8-tdTomato reporter showed expression by ~10 % of PP cDC2s, DN cDCs and Lyz DC/Mac (Supplementary Fig. 3A, B). Treatment of control (GPR35^+/–^) mice with an Itgb8 (αvβ8) integrin blocking antibody for 3 weeks starting at 3 weeks of age led to a reduction in PP IgA^+^ GC B cell and plasma cell frequencies (Supplementary Fig. 3C, D). Importantly, anti-Itgb8 treated control and GPR35 KO mice had similarly low frequencies of IgA^+^ cells (Supplementary Fig. 3C, D). Itgb8 blocking also led to reduced IgA coating of fecal bacteria in control mice (Supplementary Fig. 3E). The similarly low IgA^+^ cell frequencies and IgA coating of fecal bacteria in anti-Itgb8 treated control and GPR35 KO mice is consistent with αvβ8 functioning in the same pathway as GPR35. To examine the impact of GPR35-deficiency on Itgb8-expressing cDCs, we crossed GPR35^−/−^ and Itgb8-tgTomato reporter mice. Flow cytometric analysis of these mice revealed a major depletion of Itgb8-expressing cDC2s in the absence of GPR35 (Supplementary Fig. 3F–H). Taken together, the data are in accord with GPR35 being required for the function of Itgb8^+^ cDC2s in supporting IgA switching.

### GPR35 in cDCs is required for PP and intestinal IgA

We next performed further experiments to test the importance of GPR35 selectively in cDCs for supporting PP IgA responses. Ablation of cDCs by treating Zbtb46-DTR^+^ BM chimeras with diphtheria toxin (DT) led to a loss of PP cDCs but not Lyz DC or Lyz Mac (Supplementary Fig. 4A–C). This treatment led to a decrease in the IgA^+^ GC response but also caused a non-significant reduction of IgG1^+^ GC cells, likely due to the near complete loss of cDCs rather than selective loss of SED cDC2s (Supplementary Fig. 4D).

Previous work (5) established a prominent requirement for LTβR signaling in PP cDC2 and DN cDC homeostasis and we confirmed that finding here using LTβR^f/f^ CD11cCre mice (Supplementary Fig. 4E, F). Importantly, numbers of cDC1s, Lyz DC and Lyz Mac were not affected (Supplementary Fig. 4G–I). We therefore generated mixed BM chimeras between LTβR^f/f^ CD11cCre and WT or GPR35 KO BM to test the cDC intrinsic role of GPR35 in PP IgA responses. In these BM chimeras that lack GPR35 in most cDC2s and DN cDCs there was a reduced frequency of IgA^+^ and increased frequency of IgG1^+^ PP GC B cells, a reduced frequency of PP plasma cells, and a reduced frequency of IgA coated fecal bacteria (Supplementary Fig. 4J–L). To test the contribution of PP Lyz DC and Lyz Mac to the IgA response we took advantage of the finding that these monocyte derived cells, but not cDCs, are CCR2 dependent (21). CCR2 deficiency led to a reduction in BST2^+^ CD11c^+^ MHC2^+^ Lyz DC/Mac but not cDCs (Supplementary Fig. 4M, N) and did not alter IgA^+^ and IgG1^+^ GC B cell frequencies or IgA^+^ plasma cells (Supplementary Fig. 4O, P). These findings indicate that GPR35 function in LTβR-dependent cells, most likely cDC2s, supports the PP and intestinal IgA response.

### Tph1^+^ mast cells promote cDC2 positioning in SED and the IgA response

Mast cells have been implicated as a source of the GPR35 ligand 5-HIAA at sites of inflammation (11, 13, 22). Moreover, mast cells can augment intestinal IgA responses during inflammation (23). We therefore investigated the impact of mast cell deficiency on the PP IgA response. Examination of PPs from Kit/v × Kit/W mice that are hypomorphic for the tyrosine kinase receptor Kit and lack mast cells (24) showed a deficiency in cDC2s, but not cDC1s or DN DCs (Supplementary Fig. 5A–C). In accord with the role of cDC2s in supporting IgA responses, these mice had reduced PP IgA^+^ GC cells and CD138^+^ plasma cells (Supplementary Fig. 5D–G).

**Figure 4.**
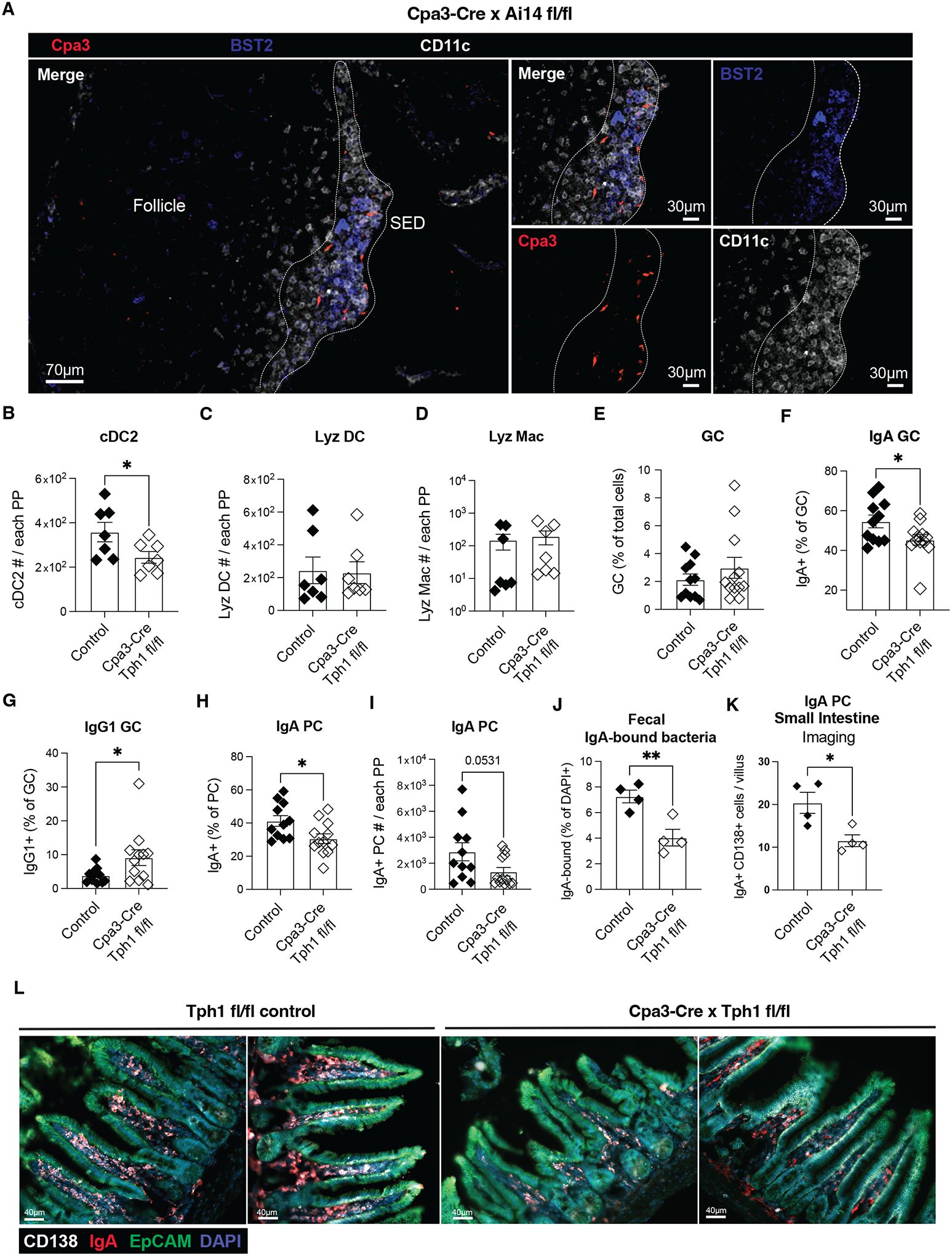
Mast cell-derived serotonin metabolite(s) support cDC2 maintenance and localization within SED to boost intestinal IgA responses. **A.** Epifluorescence micrographs of PPs from Cpa3-Cre × Ai14^fl/fl^ (mast cell reporter) mice. Cpa3 (red), BST2 (blue) and CD11c (white) signals and staining are shown. The white dashed lined delimits the SED and was depicted based on CD11c positivity. Images are representative of at least 4 mice and 2 independent experiments. **B-D**. Quantification of cDC2 (**B**), Lyz DC (**C**) and Lyz Mac (**D**) absolute numbers in PPs from control (Cpa3-Cre Tph1^fl/wt^ and Tph1^fl/fl^) and Cpa3-Cre Tph1^fl/fl^ mice. *n*=7. Data are pooled from 2 independent experiments. **E-J**. Quantification of (**E**) GC cells % (CD19^+^IgD^−^CD38^−^GL7^+^CD95^+^ out of total cells), (**F**) IgA^+^ GC cell % and (**G**) IgG1^+^ GC cell % (out of CD19^+^ GL7^+^ CD95^+^ CD38^−^ IgD^−^), (**H**) IgA^+^ plasma cells % (out of CD19^+^ IgD^−^ CD138^+^), (**I**) IgA^+^ plasma cells numbers, and (**J**) IgA-bound fecal bacteria % (out of DAPI+) in control (Cpa3-Cre Tph1^fl/wt^ and Tph1^fl/fl^) and Cpa3-Cre Tph1^fl/fl^ mice. *n*=11 (control, **E-I**); *n*=4 (control, **J**); *n*=12 (Cpa3-Cre × Tph1^fl/fl^, **E-I**); *n*=4 (Cpa3-Cre × Tph1^fl/fl^, **L**). Data are pooled from 3 (**E-I**) or 2 (**J**) independent experiments. Data are representative of 3 independent experiments. **K, L**. Quantification (**K**) of CD138^+^ IgA^+^ plasma cells per villus in images (**L**) from Tph1^fl/fl^ control (two examples, left) and Tph1^fl/fl^ Cpa3-Cre (two examples, right) mice. *n*=12. Data are pooled from 2 independent experiments. * p<0.05; ** p<0.005; *** p<0.0005. Data are presented as mean ± SEM. Two-tailed unpaired t-tests were performed.

To examine the distribution of mast cells in PPs we crossed Ai14 lsl-tdTomato reporter mice with Cpa3-Cre mice that express Cre selectively in mast cells and basophils (25). Immunofluorescence microscopy of PPs from these mice showed reporter+ cells selectively within the SED (Fig. 4A and Supplementary Fig. 5H). These were mast cells and not basophils based on expression of FcεR1 and lack of CD49b expression (Supplementary Fig. 5I).

We found that a portion of PP mast cells were surface positive for the degranulation marker CD63, indicating the presence of activated mast cells within PPs at steady state (Supplementary Fig. 5J). The fraction of surface CD63^+^ mast cells was reduced in germ free mice (Supplementary Fig. 5K) suggesting a role for the microbiome in promoting PP mast cell activation status. In accord with a role for CD63^+^ mast cells upstream of cDC2s, germ free mice had a reduced frequency and number of PP cDC2s (Supplementary Fig. 5L) and the SED was poorly developed (Supplementary Fig. 5M). GCs in germ free mice are markedly skewed away from IgA and towards IgG1 (26).

Mast cells in gut and skin express high amounts of Tph1 and Maob, enzymes needed for production of 5-HIAA (13, 27). Analysis of PP scRNAseq data (16) revealed mast cells as having the highest expression of Tph1 amongst the 42 cell types analyzed and they also expressed Maob (Supplementary Fig. 6A). We therefore crossed Tph1^f/f^ mice with Cpa3-Cre mice. Mast cells from Tph1^fl/fl^ Cpa3-Cre mice lost the ability to generate 5-HIAA *in vitro* (Supplementary Fig. 6B) and showed a corresponding loss in ability to support GPR35-expressing cell line migration (Supplementary Fig. 6C). 5-HIAA abundance was significantly reduced in tissue extracts prepared from PPs of Tph1^fl/fl^ Cpa3-Cre mice (Supplementary Fig. 6D). The partial reduction likely reflects the presence of intestinal microvilli containing Tph1^+^ enterochromaffin cells in the PP preparations. The extent of PP mast cell activation, measured by surface CD63 staining, was independent of Tph1 expression (Supplementary Fig. 6E).

Tph1^fl/fl^ Cpa3-Cre mice showed a reduction in the number of PP cDC2s but not cDC1s or PP Lyz DC and Lyz Mac (Fig. 4B–D and Supplementary Fig. 6F–H). Tph1^fl/fl^ Cpa3Cre mice had a reduced frequency of IgA^+^ PP GC B cells and increased frequency of IgG1^+^ GC B cells, and a reduction in IgA^+^ PP plasma cells, fecal IgA^+^ bacteria, and lamina propria plasma cells (Fig. 4E-L). Similar results were obtained when Tph1 expression was ablated in all bone marrow-derived cells, including mast cells (Supplementary Fig. 6I–M).

Analysis of tissue sections showed that cDC2 were reduced in the SED of mast cell deficient and mast cell Tph1-deficient mice (Supplementary Fig. 7A, B). In GPR35 gain-of-function mice treated with phenelzine to inhibit MAO and generation of 5-HIAA, GPR35^hi^ (GFP^hi^) B cells failed to accumulate in the SED (Supplementary Fig. 7C–E). Similarly, transferred B cells from GPR35 over-expressing mice were less able to access the SED in mice lacking Tph1 in mast cells (Supplementary Fig. 7F–H). Taken together, these findings suggest that by acting as a source of 5-HIAA, PP SED mast cells mediate recruitment or retention of GPR35^+^ cDC2s in the SED and thereby foster interactions between αvβ8^+^ cDC2s and B cells to promote the intestinal IgA response.

## Discussion

Mast cells are well known for their role in hypersensitivity, inflammation, and responses to irritants (24, 28). Here we identify a new role for mast cells as organizers of a lymphoid tissue niche. Our findings support a model where mast cell production of 5-HIAA in a Tph1-dependent manner leads to recruitment of GPR35^+^ αvβ8-expressing cDC2s to the PP SED. B cells in this zone interact with the cDC2s, leading to TGFβ1 activation and induction of IgA class switch. These mast cell-supported interactions contribute to IgA responses against intestinal pathogens and to production of IgA that binds the microbiome.

We focused on GPR35 as a candidate cDC2 chemoattractant receptor in this study because of its abundant expression in cDC2s in multiple datasets, and because of our recent *in vivo* data showing that GPR35 can act as a chemoattractant receptor for neutrophils and eosinophils (11, 29). Our findings in loss and gain of function mice are consistent with GPR35 supporting cDC2 chemoattraction to the SED. We suggest that GPR35-expressing cDC2s may similarly be attracted to mast cell-rich microenvironments in other tissues. We speculate that the reduced frequency of cDC2s in the PPs of GPR35-deficient mice and BM chimeras is a consequence of their less efficient positioning in the PP SED. However, we do not exclude that GPR35 plays additional roles in promoting cDC2 homeostasis. Lyz DC and Lyz Mac express high amounts of GPR35, yet their recruitment and accumulation in the SED of GPR35-deficient mice was intact. This may be due to other homing receptors promoting SED localization and maintenance of Lyz DC and Lyz Mac (Supplementary Fig. 1A). Futures studies are needed to analyze the positioning requirements of these cells. As shown previously and confirmed in this study, PP cDC2s, like cDC2s in other tissues, are dependent on LTβR signaling (5, 30, 31). Innate lymphoid cells type-3 (ILC3) that express LTα1β2^+^, the LTβR ligand, are present within the SED and are needed for maintenance of the cDC2 compartment (5). While a fraction of cDC2s is retained in the absence of GPR35, we found a major loss of itgb8-expressing cDC2s. Thus, we propose that GPR35 expression is key for Itgb8-expressing cDC2 homeostasis, and that GPR35 collaborates with other homing receptors to mediate the chemoattraction of cDC2s into the SED and thereby promote contacts with LTα1β2^+^ ILC3s, supporting cDC2 survival and interaction with B cells. GPR35 and 5-HIAA may influence both the recruitment and positioning of cDC2s in the SED, as evidenced by the proximity of GPR35-deficient cDC2s to the B cell follicle compared to controls in some of our imaging data. Future higher dimensional imaging studies will be needed to precisely define the role of GPR35 in regulating SED cDC2 positioning with respect to activated mast cells and follicles.

In the epithelial compartment, GPR35 is important for goblet cell function (32). It is possible that altered epithelial cell function in GPR35-deficient mice influences the IgA response. However, our finding of a similar defect in the IgA response in WT mice reconstituted with GPR35-deficient BM, as well as in BM chimeras selectively lacking GPR35 in cDCs, establishes that a major function for GPR35 in the IgA response is in the cDC compartment. In each of the GPR35-deficient conditions we studied, the impact on IgA plasma cells and IgA coating of commensal bacteria appeared greater than the impact on the IgA^+^ GC response. The fraction of microbiome-reactive IgA that is generated in T cell-dependent responses remains under investigation by the field, though several studies suggest that it is the major fraction (1, 2, 33). Moreover, the IgA response to cholera toxin is strongly dependent on T cell help (17). In a study that tracked plasma cell development in PPs using Blimp1-GFP reporter mice, some plasma cells emerged rapidly within the SED (34). It is possible that GPR35-dependent positioning of cDC2s in the PP SED supports not only induction of IgA^+^ pre-GC cells, but also GC-independent IgA^+^ plasma cells. IgA responses can also arise in isolated lymphoid follicles and related structures in the small intestine, and we do not exclude a role for GPR35 in cDC2s at these sites (35–39).

Recent work characterized Lyz DC and Lyz Mac as monocyte-derived myeloid cells present at variable frequencies in the PP SED that have a role in antigen capture and presentation to T cells (6). The role of these cells in the IgA response has not been previously explored. We found that a fraction of Lyz DC/Mac expressed αvβ8 integrin. Previous work showed PP cDC2s express Itgb8 mRNA and conditional removal of Itgb8 from CD11c^+^ cells led to reduced PP IgA responses (5). However, a study using Itgb8 reporter mice found minimal expression of Itgb8 in PP cDC2s (19). Using the same reporter line in the current study, we found that 5–17% of PP cDC2s expressed Itgb8. The basis for the discrepancy with the prior reporter study is unclear but most likely reflects differences in the microbiome between facilities leading to different extents of cDC2 Itgb8 expression. Despite the apparently similar Itgb8 expression by cDC2s and Lyz DC/Mac, we did not find any effect on IgA^+^ cell frequencies of Lyz DC/Mac loss caused by CCR2 deficiency. We suggest that Lyz DC/Mac are not able to sustain the close cell-cell contacts with B cells that are likely necessary for TGFβR engagement and induction of IgA switch (5). We note that Itgb8 is expressed by only a fraction of PP cDC2s. Studies with splenic cDCs showed that signals derived from the gut microenvironment (retinoic acid, TLR agonists, TGFβ) could lead to upregulation of Itgb8 expression by cDCs (40). Thus, the low expression at the cDC2 compartment level might be explained by dynamic expression through time and regulation by various signaling pathways.

Mast cell deficiency has been associated with reduced intestinal IgA responses though the mechanism involved has been unclear (23, 41). Prior studies relied on mice with c-Kit deficiency (41) or mast cell depletion by Diptheria toxin (DT) treatment in transgenic hosts (RMB mice) (23) to test the role of mast cells. However, Kit-deficient mice have defects in intestinal pacemaker cells and show altered intestinal peristalsis (42) and DT-treated RMB mice have concomitant depletion of basophils (23). Each of these defects might indirectly affect IgA responses. In accord with mast cells supporting IgA generation, purified mast cells co-cultured with B cells promoted IgA responses *in vitro*, and mast cell deficient RMB mice showed reduced intestinal IgA upon inflammation (23). Counterintuitively, however, mast cell deficient RMB mice had increased IgA production at steady state (23). While these data may highlight a dual role for mast cells depending on the intestinal inflammation state, excessive DT treatment (1mg/day per mouse) and concomitant basophil depletion may have obscured mast cell dependent regulation of intestinal IgA responses in this setting. By selectively removing Tph1 from mast cells in c-Kit intact DT-free systems, we have been able to reveal a role for mast cell function in an early step during intestinal IgA responses. The mechanism of 5-HIAA storage and release by mast cells is not fully defined and needs more study. We also note a limitation of our study is that while the similar phenotype of GPR35-deficient mice and mast cell Tph1-deficient mice is consistent with 5-HIAA being the relevant GPR35 ligand in PPs, we cannot exclude the possibility that mast cell Tph1-deficiency has altered (directly or indirectly) production of an alternative GPR35 ligand. Moreover, our data do not exclude that additional mast cell-independent host or microbial metabolites acting on GPR35, including but not limited to kynurenic acid and 5-HIAA, may also have an impact on this circuitry. In addition, whether the mast cell-mediated recruitment of cDC2s occurs in a continuous manner or is dependent on production of mast cell activating signals, such as might occur during feeding, will be an interesting area of future investigation. Our findings in germ free mice suggest that PP mast cell activation status and function in promoting IgA responses may in part be determined by the microbiome. Finally, widely used anti-depressants can affect serotonin and 5-HIAA intestinal levels, possibly interfering with the circuitry described here. In the future it will be of interest to establish whether chronic exposure to anti-depressants has an impact on intestinal IgA levels in mice and humans.

## Material and methods

### Study design

Our study was designed to elucidate the impact of GPR35 expression in Peyer’s patch cDCs and its influence on intestinal IgA production. To achieve this, we analyzed publicly available scRNAseq data (16) and conducted flow cytometry, imaging, and specific *in vitro* assays in the presence or absence of GPR35. Additional genetic perturbations, bone marrow chimeras, and retroviral transduction approaches were used to dissect the function of GPR35 in cDCs. The role of mast cells as a source of GPR35 ligand was explored using Tph1 floxed mice expressing a mast cell-specific Cre transgene, and similar analyses were performed as for the GPR35-deficient mice.

### Mice

C57BL/6J and BoyJ (CD45.1) mice were bred in an internal colony and 7–12-week-old mice of both sexes were used. Gpr35^−/−^ mice were obtained from EMMA (EM09677; Gpr35tm1b(EUCOMM)Hmgu) and maintained on a B6 background. CD11c-DTR-GFP mice were maintained on a B6 background. Mast cell-deficient Kit/v × Kit/W mice were obtained from Jackson Laboratories and maintained on a B6 background. *Tph1*^+/−^ and *Tph1*^−/−^ (43) BM were provided by Huaquing Wang and Waliul Khan (McMaster Univ.). Zbtb46-DTR mice (Zbtb46tm1(HBEGF)Mnz) (44) were from Michel Nussenzweig (Rockefeller Univ) on a B6 background. Tph1 floxed mice (45) were kindly provided by Gerard Karsenty (Columbia Univ.). Cpa3-Cre mice (25) were kindly provided by Paul Bryce (Northwestern Univ) and bred against Tph1 fl/fl mice. Itgb8-td-Tomato mice (19) were provided by Dean Sheppard (UCSF). B6.Cg-Gt(ROSA)26Sortm14(CAG-tdTomato)Hze Ai14 and NBSGW mice were obtained from Jackson Laboratories and maintained on a B6 background. *Rag1*^*–/–*^ (Rag1^tm1Mom^) mice were from a colony maintained by Averil Ma (UCSF). 8–12-week-old co-caged littermate and age and sex-matched controls (WT and HET) were used for experiments, mice were allocated to control and experimental groups randomly, sample sizes were chosen based on previous experience to obtain reproducible results and the investigators were not blinded. In experiments with chimeric mice and adoptive transfers, age and sex-matched 8–12-week-old donors and recipient mice were utilized.

### Cell transfer, treatments, immunizations, and bone marrow chimeras

For overexpressing splenocyte transfer, 3–5 × 10^7^ spleen cells from GPR35-GFP transduced BM chimeras were labeled with Deep Red (#C34565, Life Tech) according to manufacture instructions and adoptively transferred into B6, Tph1^fl/fl^ or Cpa3-Cre × Tph1^fl/fl^ recipients and mice were analyzed at the indicated time. For anti-Itgb8 treatment, 3-week-old mice were treated with neutralizing antibody by i.v. injection of 10 mg/kg of antibody every 3.5 days for 7 days. For cholera toxin immunization, adult mice were gavaged 3 times orally with 10 ug of cholera toxin (EMD Bioscience), oral immunizations were performed 7 days apart and mice were analyzed 7 days after the final immunization. To deplete total generation of 5-HIAA, mice were treated with one i.p. doses of Phenelzine (30mg/kg, #P6777, Millipore Sigma) 24hr before the experiment. To produce mixed chimeras, CD45.1 congenic Boy/J mice were lethally irradiated with 1300 rad in split doses and reconstituted with 5 × 10^6^ BM cells (~50:50) as indicated. Mice were analyzed 7-8 weeks later. Since the hematopoietic stem cell content in BM from different donor mice may not be identical, reconstitution of the B220^+^ B cell compartment was used to assess chimerism after reconstitution. B cells lack GPR35 expression and are not expected to be affected by GPR35-deficiency. To deplete Zbtb46-DTR^+^ cells, chimeric mice were treated i.p. with a first dose of 20ng/g DT (Sigma), and subsequent 4ng/g doses (every other day) for 2 weeks, starting from 6 weeks after BM reconstitution. Depletion efficiency was tested by flow cytometry. Animals were housed in a pathogen-free environment in the Laboratory Animal Resource Center at the University of California, San Francisco, and all experiments conformed to ethical principles and guidelines that were approved by the Institutional Animal Care and Use Committee.

### Generation of GPR35-expressing WEHI-231 cells

Murine and human GPR35 were cloned into the murine stem cell virus (MSCV)-GFP retroviral vector (mGPR35-GFP). The retroviruses encoding mGPR35-GFP were produced using the Platinum-E packaging cell line, as previously described (11, 46). Briefly, 5 × 10^5^ WEHI-231 cells were placed in a 6-well plate along with the retroviral supernatant and the cells were centrifuged at 1,340g (2400 rpm) for 2 h at room temperature. This spinfection was repeated with fresh retrovirus for a second time 24 h later. Then, 48 h after the second spinfection, the highest 3% of GFP-expressing cells were sorted using a BD FACSAria II.

### Transwell migration assay and mast cell purification/activation

*GPR35*^+/+^(CD45.1^+^) and *GPR35*^−/−^ (CD45.2^+^) PPs were incubated for 40 min shaking (1000rpm) at 37 °C in 1ml of digestion medium (RPMI, 1% NBCS, 0.25 mg ml−1, Liberase TM Research Grade (Sigma), 0.025 mg ml−1 DNasel (Sigma)). To stop the digestion, 100μl of quenching solution (RPMI, 50% NBCS) were added to each sample. The digested patches were then mashed through a 100-μm strainer (Fisher Scientific) and washed 3x with migration medium (RPMI containing 0.5% fatty acid-free BSA, 10 mM HEPES and 50 IU penicillin/streptomycin). The cells were resuspended in migration medium at 5–10 × 10^6^ cells / ml and resensitized for 20 min in a 37 °C water bath in migration plus medium. Transwell filters (6 mm insert, 5 μm pore size, Corning) were placed on top of each well, and 100 μl containing 2–5 × 10^5^ cells was added to the transwell insert. The cells were allowed to migrate for 3 hr, after which the cells in the bottom well were counted by flow cytometry. Representative experiments for each migration assay are plotted as a percentage of input migration. Mast cells were isolated from peritoneal lavage of B6 mice by positive selection (EasySep Mouse CD117 (cKIT) Positive Selection Kit), as previously described (De Giovanni et al., 2022). Isolated primary mast cells were diluted in migration medium and seeded in flat 24 well plates (5×10^5^ cells / well) 30 min before activation with LPS (100ng/ml, Sigma). Supernatants were collected 2hr after activation, centrifuged at 10000 rpm for 30min at 4°C and tested for migration or ELISA. To quantify 5-HIAA in cell supernatants, Mouse 5-Hydroxyindoleacetic acid (5-HIAA) ELISA Kit (AssayGenie) was used following manufacturer instructions.

### Fecal total IgA ELISA

Fecal pellets were collected from mice and weighed prior to processing. One to two pellets were collected per mouse and mice were placed in glass beakers simultaneously for consistent collection of the first two available pellets. Pellets were smashed through a 100 μm filter in 1X Cell Lysis Buffer (Cell Signaling Technology) with 1 mM PMSF at a ratio of 100 μl to 10 mg of feces. The strainer and collection dish were then washed with an additional 1ml of PBS+1%BSA. Processed fecal pellets were spun at 4C for 13,000g for 10 min. Supernatants were collected and stored at −70C until analysis. For total IgA detection, ELISA plates (Corning Costar 9018) were coated with 1 μg/ml goat anti-mouse IgA (IgA mouse ELISA kit, Invitrogen) capture antibody overnight at 4C. Plates were washed and blocked with 1% BSA, 0.1% Tween in PBS overnight at 4C. Each sample was serially diluted 5x from a starting dilution of 320x, with at least one technical replicate for each dilution. Standards were 2x serial dilutions between 25 and 0.78 ng/ml. Diluted samples and standards were added and incubated for 2 hours at RT. After incubation, plates were washed to remove unbound antibodies prior to incubation with HRP-conjugated goat anti-mouse IgA antibody for 1 hour at RT to detect captured IgA. ELISA plates were developed by TMB microwell peroxidase substrate for 15 min and quenched by 1 M HCl. Colorimetric reaction was measured at OD = 450 nm, subtracting the OD at 570 nm. The OD values that fell within an acceptable range of the standard curves were used to interpolate a ng/ml concentration for each sample. The final ng/ml concentration for each sample is the average of the interpolated values and their technical replicates, multiplied by the sample dilution factor. Total IgA content was then normalized to the weight of collected feces.

### CT-specific IgA ELISA.

CT-specific IgA quantification by ELISA was as described (5). Briefly, ninety-six-well plates (Thermo Fisher Scientific) were coated with purified anti-IgA (RMA-1, BD) or 0.5nM ml GM1 followed by 0.5 μg/ml CT overnight at 4 °C (47). The plates were washed and clocked with PBS/5% BSA before diluted fecal samples were added. Samples were incubated overnight at 4 °C, followed by biotinylated anti-mouse antibodies: anti-IgA (C10–1, BD) at 1 ug/ml in PBS/0.1% BSA. Detection antibodies were labeled by streptavidin-conjugated horseradish peroxidase (HRP) and visualized by the addition of Substrate Reagent Pack (R&D). Color development was stopped with 3M H2SO4. Absorbances at 450 nm were measured on a tunable microplate reader (VersaMax, Molecular Devices). Single dilutions (1:3–1:10) were analyzed for each of the samples. CT-specific antibody relative levels were calculated by extrapolating absorbance values multiplied for the dilution factor used for each of the samples, using SoftMax Pro 5 and Prism software.

### Generation of over-expressing bone marrow chimeras

Mice to be used as BM donors (C57BL/6J) were injected intravenously with 3 mg 5-fluorouracil (Sigma). BM was collected after 4 days and cultured in DMEM containing 15% (v/v) FBS, antibiotics penicillin (50 IU/ml) and streptomycin (50mg/ml); Fisher) and 10 mM HEPES, pH 7.2 (Cellgro), supplemented with IL-3 (#213–13, Peprotech), IL-6 (#216–16, Peprotech and stem cell factor (#250–03, Peprotech) at concentrations of 20, 50 or 100 ng/ml, respectively). Cells were spin-infected twice at days 1 and 2 with viral supernatant (MSCV-GPR35-IRES-eGFP or the empty vector (EV) that lacked the GPR35 insert) and transferred into irradiated CD45.1 B6 recipient mice on day 3, similarly to what was previously described (47).

### Flow cytometry

cDC2 were identified as CD11c^+^ Mertk^−^ MHC2^+^ CD8^−^ CD11b^+^; cDC1 were identified as CD11c^+^ Mertk^−^ MHC2^+^ CD8^+^ CD11b^−^; DN were identified as CD11c^+^ Mertk^−^ MHC2^+^ CD8^−^ CD11b^−^; Lyz DC were identified as CD11c^+^ BST2^+^ MHC2^+^ CD4^−^; Lyz Mac were identified as CD11c^+^ BST2^+^ MHC2^+^ CD4^+^. Cells were stained with the following antibodies. BV605-conjugated rat anti-mouse CD45.2 (104, #109841, BioLegend); Pacific Blue-conjugated rat anti-mouse IgD (11–26c.2a, # 405712; BioLegend); Pacific Blue-conjugated rat anti-mouse CD3 (17A2, #100214, BioLegend); Pacific Blue-conjugated rat anti-mouse CD19 (6D5, # 115523, BioLegend); APC-conjugated rat anti-mouse Mertk (DS5MMER, # 17-575-182, Fisher Scientific); AF488-conjugated rat anti-mouse BST2 (927, # 127012, BioLegend); PerCP/Cy5.5-conjugated rat anti-mouse MHC2 (AF6–120.1, # 116416, BioLegend); PE-Cy7-conjugated rat anti-mouse CD11c (N418, 117308, BioLegend); BV785-conjugated rat anti-mouse/human CD11b (M1/70, #101243, BioLegend); PE-conjugated rat anti-mouse CD4 (RM4–5, #100511, BioLegend). For mixed chimeras experiments the following additional antibodies were used to discriminate between WT and KO cells: BV605-conjugated rat anti-mouse CD45.2 (104, #109841, BioLegend) and AF700-conjugated rat anti-mouse CD45.1 (A20, 110724, BioLegend). GC B cells were identified as CD19+ IgD- CD38- GL7+ CD95+ cells; IgA plasma cells were identified as CD19+ IgD- CD138+ IgA+ (PPs) or CD98high IgA+ (Lamina Propria). Cells were stained with the following antibodies. BV605-conjugated rat anti-mouse CD19 (6D5, #115540, BioLegend); Pacific Blue-conjugated rat anti-mouse IgD (11–26c.2a, # 405712; BioLegend); AF647-conjugated rat anti-mouse GL7 (Ly-77, #144606, BioLegend); PE-Cy7-conjugated rat anti-mouse CD95 (JO2, #557653, Fisher Scientific); PE-conjugated anti-mouse IgA (#1040–09, Fisher Scientific); FITC-conjugated anti-mouse IgG1 (A85-1, #553443, Fisher Scientific); PE-conjugated anti-mouse IgG2b (RMG2b-1, #406708, BioLegend); APC-conjugated rat anti-mouse CD138 (281–2, # 142526, BioLegend); BV711-conjugated CD98 (#745466, Fisher Scientific); AF421-conjugated rat anti mouse CD45.2 (104, # 109832, BioLegend); BV605-conjugated rat anti-mouse CD45.2 (104, #109841, BioLegend) and AF700-conjugated rat anti-mouse CD38 (90, #102718, BioLegend). Mast cells were stained with FITC-conjugated rat anti-mouse CD63 (NVG-2, #143920, BioLegend); PE-conjugated rat anti-mouse CD117 (2B8, #553355, Fisher); APC anti-mouse FcεRIα Antibody (MAR-1, #134316; Biolegend). For flow cytometry analysis, digested PPs were prepared as for Transwell assays. Spleens were mashed through a 100-μm strainer 70-μm in RPMI, 1%NBCS, 0.1m EDTA. Small intestine samples were processed with Lamina Propria dissociation kit (Miltenyi Biotec) and stained as indicated. Lamina Propria plasma cells were identified as CD45^+^ IgA^+^ CD98high cells. Rabbit polyclonal anti-GPR35 was produced by Biomatik (using as immunogen the C-terminus peptide: MAREFQEASKPATSSNTPHKSQDSQILSLT) and affinity-purified. To reduce non-specific background, anti-GPR35 polyclonal antibody was pre-absorbed against GPR35 KO lung cells overnight at 4°C. Cells were surface-stained, fixed and permeabilized (eBioscience^™^, #00552100) before intracellular staining. AF647-Goat anti-Rabbit IgG (H+L) Highly Cross-Adsorbed Ab (A21245, Fisher Scientific) was used as secondary antibody. Examples of the flow cytometry gating strategies used in this manuscript are show in Supplementary Figure 8A–G. Data were acquired using a BD LSR II flow cytometer or a Cytek Aurora. Flow cytometry data were analyzed using Flowjo (v.10.6.2).

### Flow cytometric analysis of IgA-bound bacteria.

Flow cytometric analysis of gut bacteria in feces was as described (5). Briefly, fecal or small intestinal pellets were suspended in filtered PBS (100 μl to 10 mg feces), homogenized well and centrifuged at 400 g for 5 min to remove larger particles from the fecal suspension. Supernatant containing bacteria was centrifuged at 8000 g for 10 min. The bacterial pellet was blocked on ice in 1 ml of BSA/PBS (1 % w/v) for 15′. Samples were spun at 8000 g for 10 min. Bacteria were stained with anti-IgA-PE on ice for 20 min and washed with PBS. Finally, bacterial pellets were stained with DAPI (AbCam) and analyzed using a Cytek Aurora or an LSRII flow cytometer.

### Whole-mount imaging, immunofluorescence and image analysis.

Whole-mount imaging was done as previously described (34, 48). Briefly, PPs were fixed in 0.05 M phosphate buffer containing 0.1 M L-lysine (pH 7.4), 2 mg/ml NaIO4 and 1 % PFA overnight at 4°C. Tissues were then blocked and permeabilized for at least 8hrs at 37°C. Samples were stained with fluorochrome or biotin conjugated antibodies in blocking buffer for 2–3 days at 37°C and then washed overnight. PE-conjugated anti-CD45.1 (A20, # 110708, BioLegend); biotin-conjugated anti-CD45.2 (104, # 109804); AF647-conjugated anti-CD11c (N418, #117312, BioLegend) and Streptavidin BV421 (#405226, BioLegend) were used. Follicle and subepithelial dome (SED) were defined based on BST2, IgD and/or CD11c positivity as indicated. To identify CD45.1 (WT) and CD45.2 (GPR35^−/−^) cDCs and Lyz DC/Mac, CD11c^+^ cells were masked using IMARIS v.9.6.0 built-in surface function. Total CD11c^+^ cells were than divided into CD45.1^+^CD452-BST2- WT cDC; CD45.1^−^CD452^+^CD11c^+^BST2^−^ KO cDC; CD45.1^+^CD452^−^BST2^+^ WT Lyz DC/Mac or CD45.1^−^CD452^+^BST2^+^ WT Lyz DC/Mac. 30–40 z stacks of 3μm each were acquired on Leica SP8-Stellaris confocal microscope. For immunofluorescence, PPs or ileum were fixed in PBS PFA 1% for 1–2hr and dehydrated overnight in 30% sucrose solution at 4°C. Tissues were than incubated and frozen in OCT and 10μm cryostat slices were obtained. Tissue slices were blocked and permeabilized in blocking buffer (PBS 0.2% Triton X-100 BSA 5%) for 1hr at RT, and then stained with the following primary antibodies: AF647-conjugated anti-CD11c (N418, #117312, BioLegend); AF488-conjugated rat anti-mouse BST2 (927, # 127012, BioLegend); PE-conjugated anti-CD11b (M1/70, # 101208, BioLegend); Pacific Blue-conjugated rat anti-mouse IgD (11–26c.2a, # 405712; BioLegend); APC-conjugated anti-mouse CD138 (281-2; #142506; BioLegend); Biotin-conjugated anti-mouse CD326 (Ep-CAM) (G8.8; #118204; BioLegend); PE-conjugated anti-mouse IgA (#1040–09, Fisher Scientific) for at least 2hr at RT in staining buffer (PBS 0.1% Triton X-100 BSA 0.5%). Samples stained with anti-mouse CD326 (Ep-CAM) were washed (2x) and incubated with FITC-conjugated streptavidin (#554061, Fisher Scientific) for 1hr at RT. Where indicated, samples were counterstained with DAPI for 10–15 minutes. CD138^+^ IgA^+^ plasma cells per villus were manually quantified. DAPI staining was used to accurately identify single cells. A mean value for each mouse was obtained after quantification of 10–15 independent snapshots. GFP-high and GFP-low cells were identified as the 30% highest or 30–40% lowest GFP^+^ cells within B220^+^ CD11c^–^ cells in each image, respectively. Images were captured on a Zeiss AxioObserver Z1 inverted microscope. All images were analyzed with Imaris v.9.6.0.

### Single-cell RNA sequencing data

We interrogated a published and pre-analyzed scRNAseq dataset of PP cells (16). The data were analyzed using the reported portal (https://singlecell.broadinstitute.org).

### Quantification and statistical analysis

Prism software (GraphPad 9.0.1) was used for all statistical analyses. The statistical tests used are specified in the figure legends. Two-tailed unpaired t-tests were performed when comparing only two groups, Paired- t-tests were used to compare internally controlled replicates, and ordinary one-way ANOVA using Turkey’s multiple comparisons test was performed when comparing one variable across multiple groups. P < 0.05 was considered significant. In summary graphs, points indicate individual samples and horizontal lines are means or medians as indicated. In bar graphs, bars show means and error bars indicate standard error mean (SEM).

## Supplementary Material

Supplementary Movie 1 Supplementary movie 1.
3D reconstruction of whole-mount SED imaging showing CD45.1 (red), CD45.2 (blue), CD11c (white) and BST2 (green) staining (left); or cDC WT (red), cDC KO (blue), BST2 (green) gated populations (right), from CD45.1 WT/ CD45.2 GPR35^+/−^ control mixed chimeras.

Supplementary Movie 2 Supplementary movie 2.
3D reconstruction of whole-mount SED imaging showing CD45.1 (red), CD45.2 (blue), CD11c (white) and BST2 (green) staining (left); or cDC WT (red), cDC KO (blue), BST2 (green) gated populations (right), from CD45.1 WT/ CD45.2 GPR35^−/−^ mixed chimeras.

Supplementary Data file S1

Supplementary Figures 1-7

## Data Availability

All data needed to evaluate the conclusions in the paper are present in the paper or the Supplementary Materials. The analyzed public scRNAseq dataset interrogated (16) is available online at: (https://singlecell.broadinstitute.org).
