# Supplementary material for: Mast cells help organize the Peyer’s patch niche for induction of IgA resonses
Source: Sci Immunol. Author manuscript; Available in PMC 2024 Apr 11. (PMC11008922; doi:10.1126/sciimmunol.adj7363)
Supplement: Supplementary Figures 1-7 [file NIHMS1973676-supplement-Supplementary_Figures_1-7.pdf]

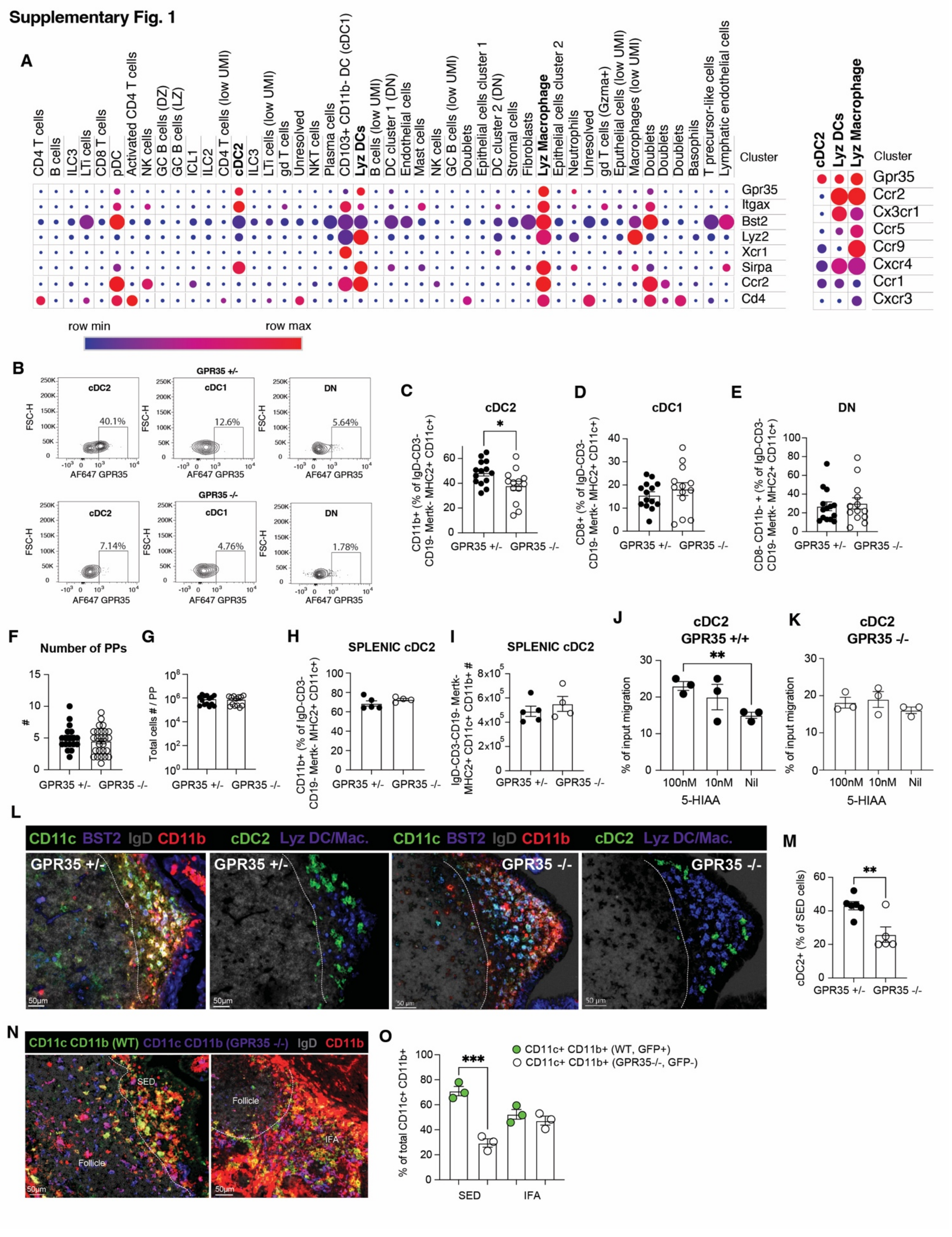

Supplementary Figure 1. GPR35 expression supports cDC2 maintenance and localization within SED. A. Dot plot showing gene expression levels in PP cell clusters in scRNAseq data

adapted from *Xu et al., (16)* (Single Cell Portal). Low (blue) and high (red) expression levels are indicated for each gene. Dot size directly correlates with % of expressing cells. **B**. GPR35 intracellular flow cytometry staining in cDC2, cDC1 and DN from $GPR35^{+/-}$ (top) and $GPR35^{-/-}$ (bottom) mice. Data are representative of 2 independent experiments. **C-E**. Quantification of cDC2 (**C**), cDC1 (**D**) and DN DC (**E**) % (out of total cDC) in PPs from $GPR35^{+/-}$ and $GPR35^{-/-}$ mice. *n*=14 ($GPR35^{+/-}$); *n*=13 ($GPR35^{-/-}$). Data are pooled from 3 independent experiments. **F, G**. Quantification of PP number (**F**) and PP total cells (**G**) in $GPR35^{+/-}$ and $GPR35^{-/-}$ mice. *n*=18, $GPR35^{+/-}$ F; *n*=26, $GPR35^{-/-}$ F; *n*=14, $GPR35^{+/-}$ G; *n*=13, $GPR35^{-/-}$ G. **H, I**. Quantification of splenic cDC2 % (**H**) and absolute numbers (**I**) from $GPR35^{+/-}$ and $GPR35^{-/-}$ mice. *n*=5 (GPR35 $^{+/-}$); *n*=4 ($GPR35^{-/-}$). Data are pooled from 2 independent experiments. **J, K**. Quantification of $GPR35^{+/+}$ (**J**) and $GPR35^{-/-}$ (**K**) PP cDC2 in vitro migration to 5-HIAA or no chemoattractant (Nil) at the indicated concentrations. Each dot represents a technical replicate (*n*=3). Data are representative of 3 independent experiments. **L**. Immunofluorescence micrographs showing PPs from $GPR35^{+/-}$ (left) and $GPR35^{-/-}$ (right) mice. CD11c (green), BST2 (blue), CD11b (red) and IgD (grey) staining (left images) or cDC2 and Lyz DC/Lyz Mac gated populations (right images) are shown. SED, subepithelial dome. White dashed delimits the SED and was depicted based on BST2 and IgD staining. **M**. Quantification of cDC2 % in SED from $GPR35^{+/-}$ (*n*=6) or $GPR35^{-/-}$ (*n*=5) mice, from images shown in **L**. Data are pooled from 2 independent experiments. **N, O**. Immunofluorescence micrographs (**N**) and quantification (**O**) of $CD11c^{+}$ $CD11b^{+}$ WT (GFP^{+}) or $GPR35^{-/-}$ (GFP^{-}) cells within SED and IFA of PPs from CD11c-GFP/ $GPR35^{-/-}$ mixed chimeras. *n*=3. Data are pooled from 2 independent experiments. * $p<0.05$; ** $p<0.005$; *** $p<0.0005$. Data are presented as mean ± SEM. Two-tailed unpaired t-tests were performed (**C-I; M**) and ordinary one-way ANOVA using Turkey's multiple comparisons test were performed (**J, K, O**).

Supplementary Fig. 2

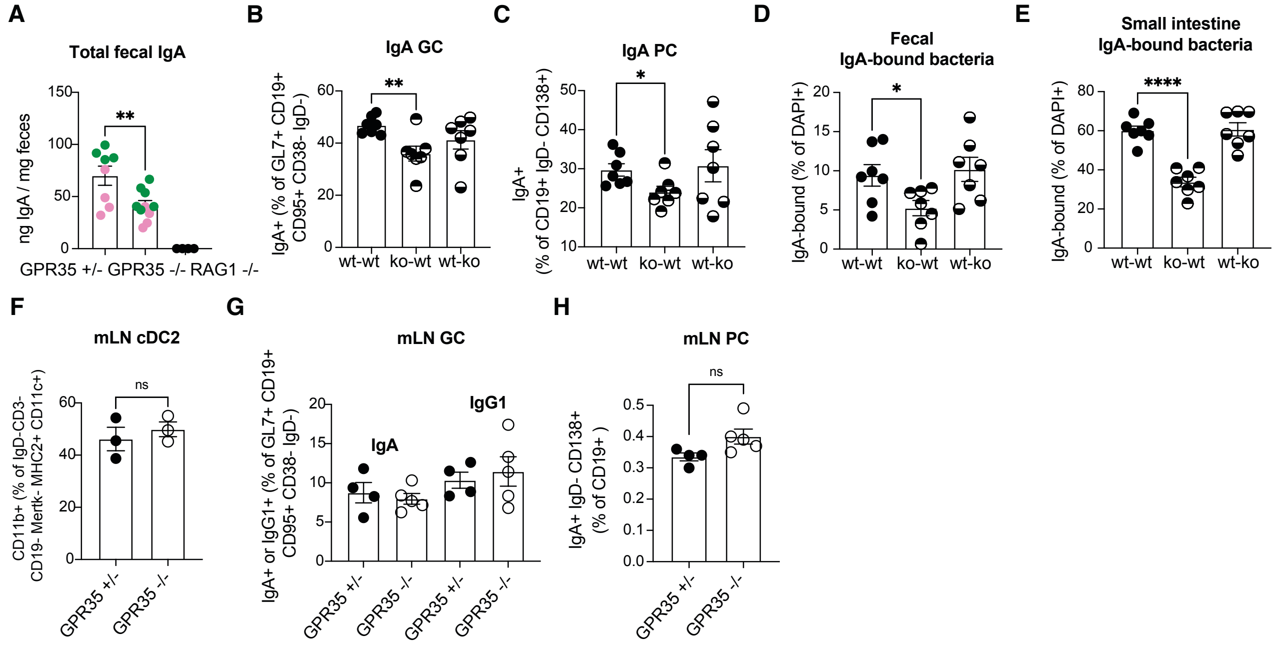

Supplementary Figure 2. Bone marrow derived GPR35-expressing cells support intestinal IgA responses. A. ELISA quantification of fecal total free IgA in GPR35$^{+/-}$ (*n*=9), GPR35$^{-/-}$ (*n*=9) and negative controls (RAG1$^{-/-}$, *n*=4). Data are pooled from 2 independent experiments (distinguished by color). **B, C**. Quantification of IgA^{+} GC % (out of GL7^{+} CD95^{+} CD38^{-} IgD^{-} CD19^{+} cells, **B**) and IgA^{+} plasma cells % (out of CD19^{+} IgD^{-} CD138^{+} cells, **C**) in GPR35$^{+/+}$ BM -> GPR35$^{+/+}$ recipient (wt-wt), GPR35$^{-/-}$ BM -> GPR35$^{+/+}$ recipient (ko-wt) and GPR35$^{+/+}$ BM -> GPR35$^{-/-}$ (wt-ko) chimeric mice. *n*=7. Data are pooled from 2 independent experiments. **D, E**. Quantification of fecal (**D**) and small intestine (**E**) IgA-bound bacteria in wt-wt, ko-wt and wt-ko chimeric mice. *n*=7. Data are pooled from 2 independent experiments. **F-H.** Quantification of cDC2 % (**F**, *n*=3), IgA^{+} and IgG1^{+} GC % (**G**, *n*=4) and IgA^{+} plasma cells % (**H**, *n*=4) in mesenteric LNs from GPR35$^{+/-}$ and GPR35$^{-/-}$ mice. Data are pooled from 2 independent experiments. * $p<0.05$; ** $p<0.005$; *** $p<0.0005$. Data are presented as mean ± SEM. Two-tailed unpaired t-tests (**A, F, H**) and ordinary one-way ANOVA using Turkey's multiple comparisons test were performed (**B-E; G**).

Supplementary Fig. 3

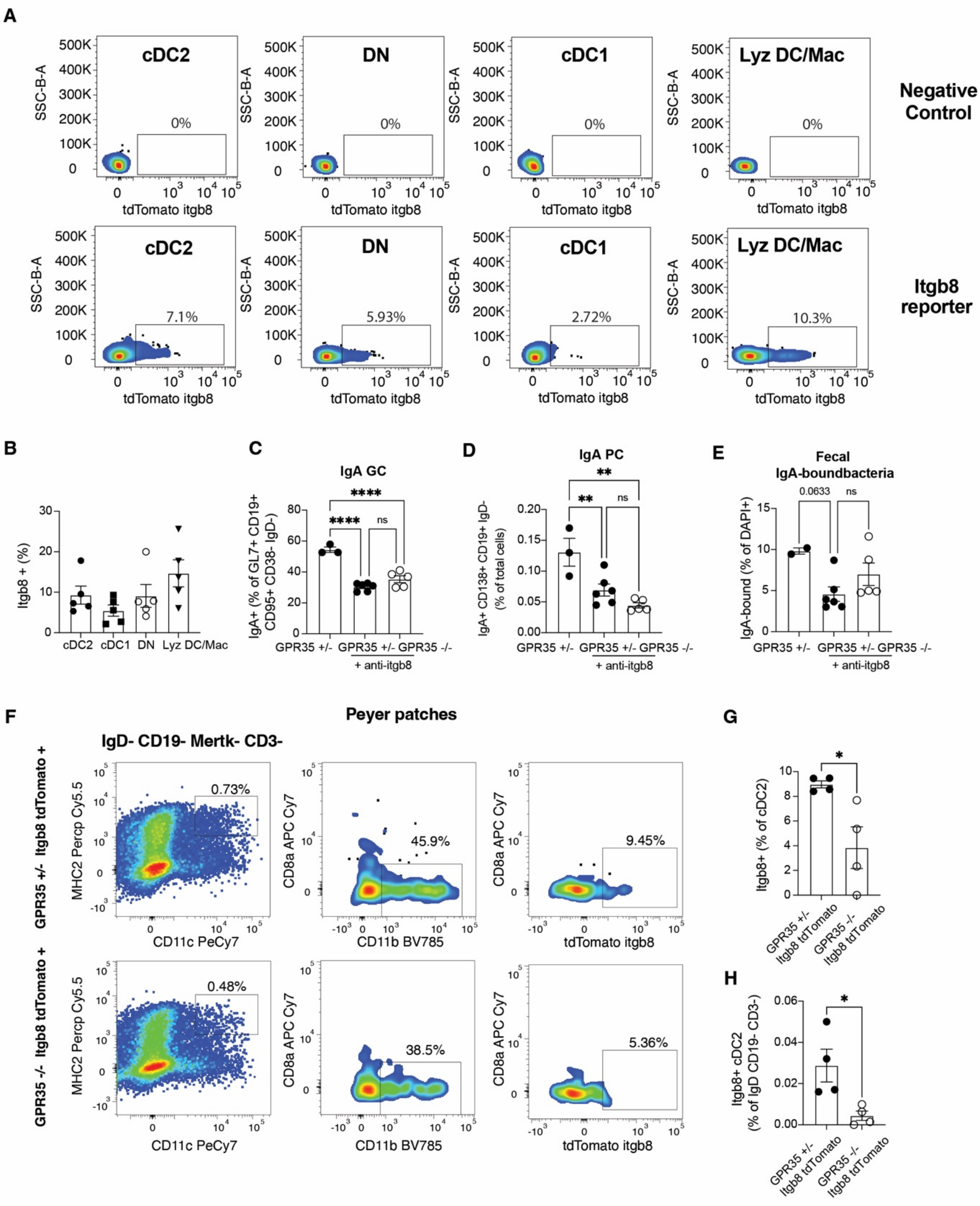

Supplementary Figure 3. GPR35 supports intestinal IgA responses via Itgb8. A, B. Representative flow cytometry plots showing itgb8 expression levels from negative control (top) and itgb8-reporter mice (bottom) within cDC2, DN DC, cDC1 and Lyz DC/Lyz Mac (**A**) and relative Itgb8 % quantification (**B**). **C-E**. Quantification of IgA^{+} GC % (out of GL7^{+} CD95^{+}

CD38$^-$ IgD$^-$ CD19$^+$ cells, **C**), IgA$^+$ plasma cells % (out of CD19$^+$ IgD$^-$ CD138$^+$ cells, **D**) and fecal IgA-bound bacteria (out of DAPI$^+$, **E**) in control mice or mice treated for 3 weeks with Itgb8 blocking antibody. *n*=3 (GPR35$^{+/+}$, **C, D**); *n*=6 (GPR35$^{+/-}$ + anti-itgb8, **C-E**); *n*=5 (GPR35$^{-/-}$ + anti-itgb8, **C-E**); *n*=2 (GPR35$^{+/+}$, E). Data are pooled from 2 independent experiments. Data are representative of 3 independent experiments. **F-H**. Representative flow cytometry plots from GPR35$^{+/-}$ or GPR35$^{-/-}$ itgb8-tdTomato reporter mice (**F**). Quantification of itgb8 % out of cDC2 (**G**) and itgb8 cDC2 % out of CD19$^-$ IgD$^-$ CD3$^-$ (**H**) of data shown in **F**. *n*=4. Data are pooled from 2 independent experiments. * p<0.05; ** p<0.005; *** p<0.0005. Data are presented as mean ± SEM. Two-tailed unpaired t-tests (**G, H**) and ordinary one-way ANOVA using Turkey's multiple comparisons test were performed (**B-E**).

Supplementary Fig. 4

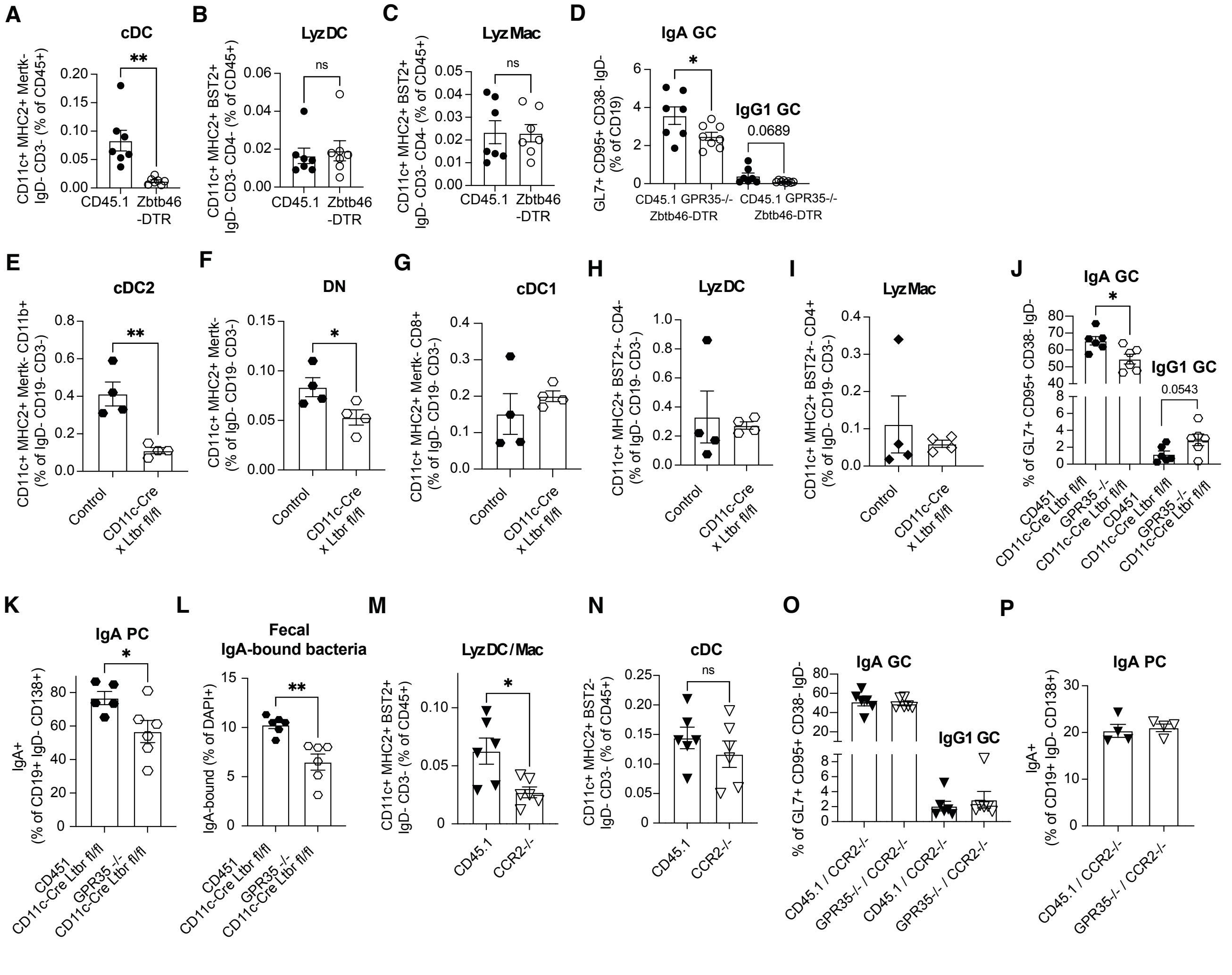

Supplementary Figure 4. GPR35-expressing cDC2 support intestinal IgA B cell responses.

A-D. Quantification of cDC (**A**), Lyz DC (**B**), Lyz Mac (**C**) % out of CD45.1^{+} (WT) or CD45.2+ (Zbtb46-DTR) cells, and IgA^{+} (left, **D**) and IgG1^{+} (right, **D**) GC % (out of CD19^{+}) within CD45.1 (WT) or GPR35$^{-/-}$ + Zbtb46 mixed chimeric mice. *n*=7. Data are pooled from 2 independent experiments. **E-I**. Quantification of cDC2 (**E**), DN DC (**F**), cDC1 (**G**), Lyz DC (**H**) and Lyz Mac (**I**) % out of IgD^{-} CD3^{-} CD19^{-} cells in control (CD11c-Cre LTbr$^{fl/wt}$ and LTbr$^{fl/fl}$) and CD11c-Cre LTbr$^{fl/fl}$ mice. *n*=4. Data are representative of 2 independent experiments. **J-L**. Quantification of IgA^{+} GC (left, **J**), IgG1^{+} GC (right, **J**), IgA^{+} plasma cells (**K**) and fecal IgA-bound bacteria in CD45.1/CD11c-Cre LTbr$^{fl/fl}$ and GPR35$^{-/-}$ / CD11c-Cre LTbr$^{fl/fl}$ mixed chimeric mice (**L**). *n*=6. Data are pooled from 2 independent experiments. **M-O**. Quantification of Lyz DC/Mac (**M**), cDCs (**N**),

IgA^{+} GC (left, **O**), IgG1^{+} GC (right, **O**) and IgA^{+} plasma cells (**P**) from CD45.1 / CCR2$^{-/-}$ and GPR35$^{-/-}$ / CCR2$^{-/-}$ mixed chimeric mice. *n*=6 (**M-O**); *n*=4 (**P**). Data are pooled from 2 independent experiments. * p<0.05; ** p<0.005; *** p<0.0005. Data are presented as mean ± SEM. Two-tailed unpaired t-tests were performed.

Supplementary Fig. 5

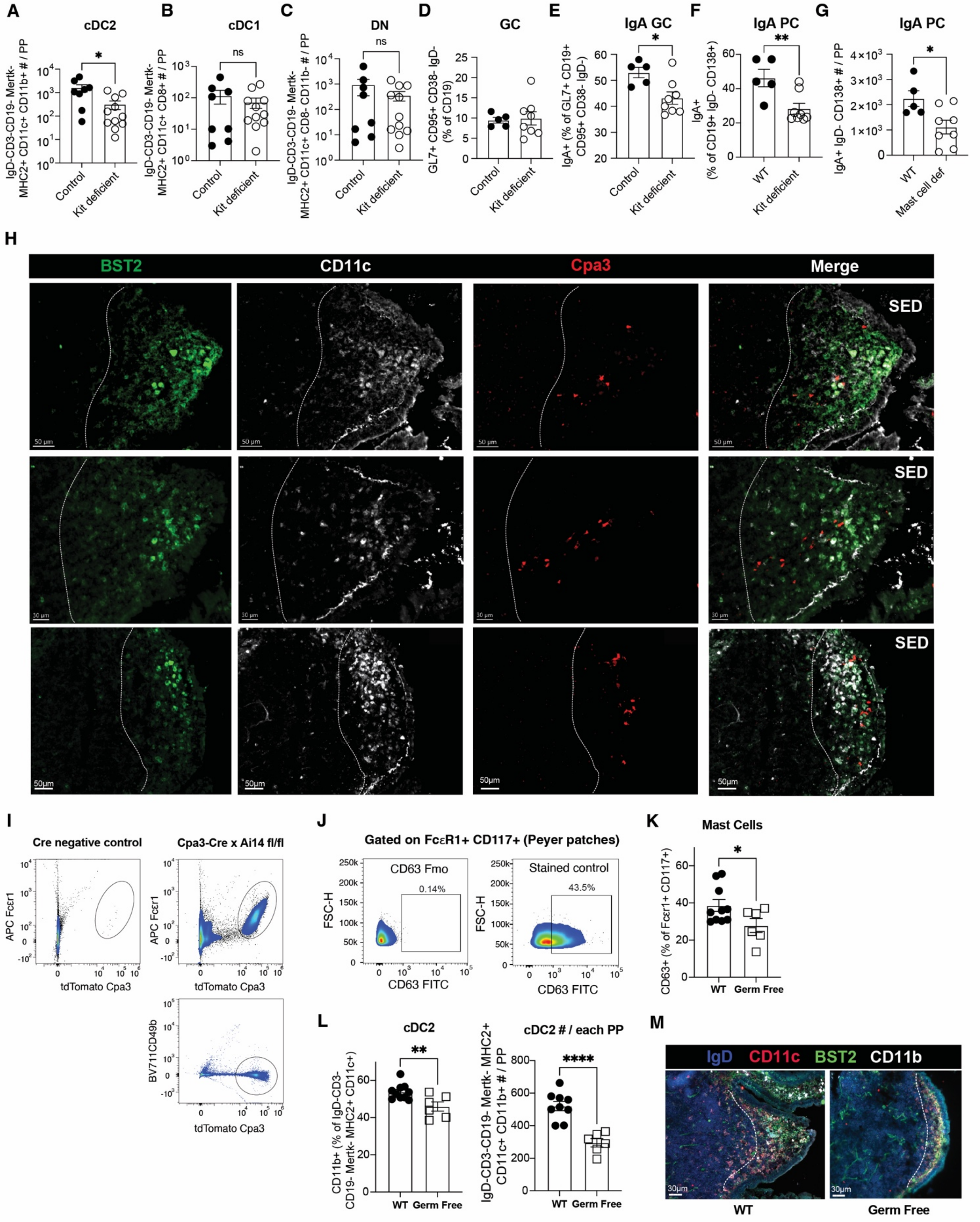

Supplementary Figure 5. Mast cells support intestinal IgA B cell responses. A-G. Quantification of cDC2 (**A**), cDC1 (**B**), DN DC (**C**), total GC (**D**), IgA^+ GC (**E**) and IgA^+ plasma cells % (**F**) and numbers (**G**) in WT and mast cell deficient (Kit/v x Kit/W) mice. *n*=8 (WT, **A-C**); *n*=5 (WT, **D-G**) *n*=11 (Mast cell-deficient, **A-C**); *n*=8 (Mast cell deficient, **D-G**). Data are pooled from 3 (**A-C**) or 2 (**D-G**) independent experiments. **H**. Immunofluorescence micrographs showing PPs from Cpa3-Cre $Ai14^{fl/fl}$ mice. BST2 (green), CD11c (white), Cpa3 (red) staining and signal are shown. SED= subepithelial dome. White dashed lines identify the SED and were depicted based on BST2, CD11c and IgD (not shown) staining. Images are representative of multiple sections from at least 4 mice and 2 independent experiments. **I**. Flow cytometry plots showing Cpa3-Ai14 (tdTomato), Fcεr1 and CD49b levels in negative control (left) and in Cpa3-Cre $Ai14^{fl/fl}$ mice**. J.** Representative flow cytometry plots of PP mast cells identified as $Fc\varepsilon R1^+CD117^+$, showing CD63 (right) and fluorescence-minus-one (Fmo) control (left). Gate used to determine % $CD63^+$ cells is shown. Data are representative of at least 2 independent experiments. **K**, **L**. Quantification of $CD63^+$ mast cell % (**K**) and cDC2s % (**L,** left) and absolute numbers (**L**, right) in germ free PPs. *n*=9 (WT); *n*=6 (germ free). Data are pooled from 2 independent experiments. **M**. Immunofluorescence microscopy of WT (left) and germ free (right) PPs to detect IgD (blue), CD11c (red), BST2 (green), and CD11b (white). Data representative of multiple sections from each of 6 mice. * $p<0.05$; ** $p<0.005$; *** $p<0.0005$. Data are presented as mean ± SEM. Two-tailed unpaired t-tests were performed.

Supplementary Fig. 6

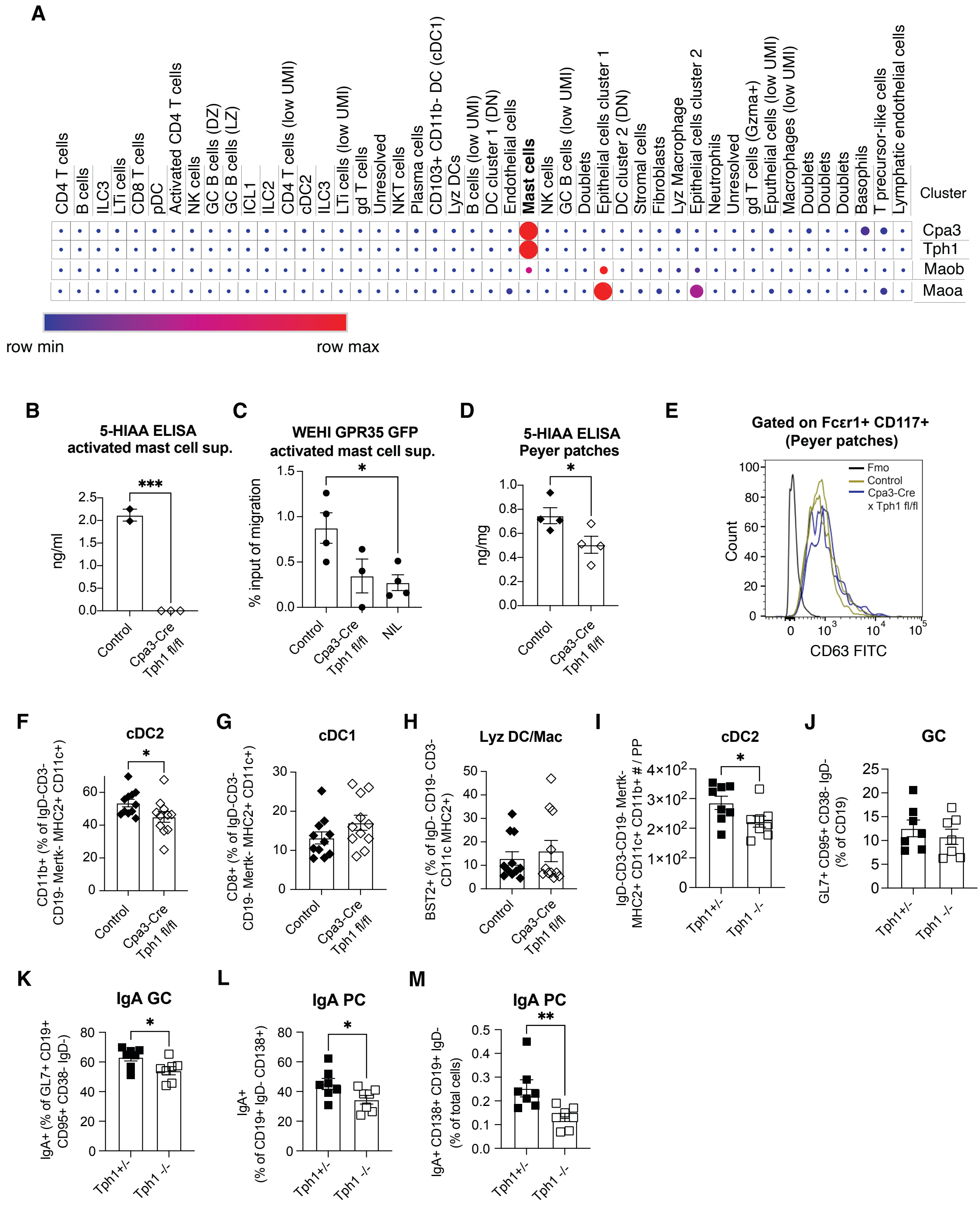

Supplementary Figure 6. Mast cell-derived serotonin metabolite(s) sustain cDC2 maintenance and localization within SED to drive intestinal IgA responses. Dot plot showing *Cpa3* and *Tph1* gene expression levels in PP cell clusters from published scRNAseq data (adapted from *Xu et al., Immunity 2019*, Single Cell Portal). Low (blue) and High (red)

expression levels are indicated for each gene. **B.** ELISA quantification of 5-HIAA in supernatants from LPS-activated Control (Tph1$^{fl/fl}$) and Cpa3-Cre x Tph1$^{fl/fl}$ peritoneal mast cells. *n*=2 (Control); *n*=3 (Cpa3-Cre x Tph1$^{fl/fl}$). **C**. Transwell migration assay with GPR35-overespressing WEHI231 cells to supernatants from Control (Tph1$^{fl/fl}$) or Cpa3-Cre Tph1$^{fl/fl}$ activated peritoneal mast cell supernatant, or to medium alone (Nil). *n*=4 (Control and Nil); *n*=3 (Cpa3-Cre Tph1$^{fl/fl}$). **D**. ELISA quantification of 5-HIAA in extracts of PPs isolated from Tph1$^{fl/fl}$ and Cpa3-Cre Tph1$^{fl/fl}$ mice. *n*=4. Each dot represents supernatants obtained from independent mast cell pools (**B**, **C**) or independent PP pool (**D**). Data are pooled from 2 independent experiments. **E**. Representative histogram overlay plot of surface CD63 on Fcεr1$^+$CD117$^+$ PP mast cells in control and Cpa3-Cre Tph1$^{fl/fl}$ mice (n=2 of each). Fmo, fluorescence-minus-one refers to sample stained without CD63 antibody. **F-H**. Quantification of cDC2 (**F**), cDC1 (**G**) and LyZ DC/Mac (**H**) in PP from control (Cpa3-Cre Tph1$^{fl/+}$ and Tph1$^{fl/fl}$) and Cpa3-Cre Tph1$^{fl/fl}$ mice. *n*=11 (control); *n*=12 (Cpa3-Cre x Tph1$^{fl/fl}$). Data are pooled from 3 independent experiments. **I-M**. Quantification of cDC2 absolute numbers (**I**), total GC % (**J**), IgA$^+$ GC % (**K**) and IgA$^+$ plasma cells % (out of total plasma cells, **L**; out of total cells, **M**) in PPs from Tph1$^{+/-}$ and Tph1$^{-/-}$ chimeric mice. *n*=8 (**I**); *n*=7 (**J-M**). Data are pooled from 3 independent experiments. * $p<0.05$; ** $p<0.005$; *** $p<0.0005$. Data are presented as mean ± SEM. Two-tailed unpaired t-tests (**B-D; F-M**) and ordinary one-way ANOVA using Turkey's multiple comparisons test were performed (**C**).

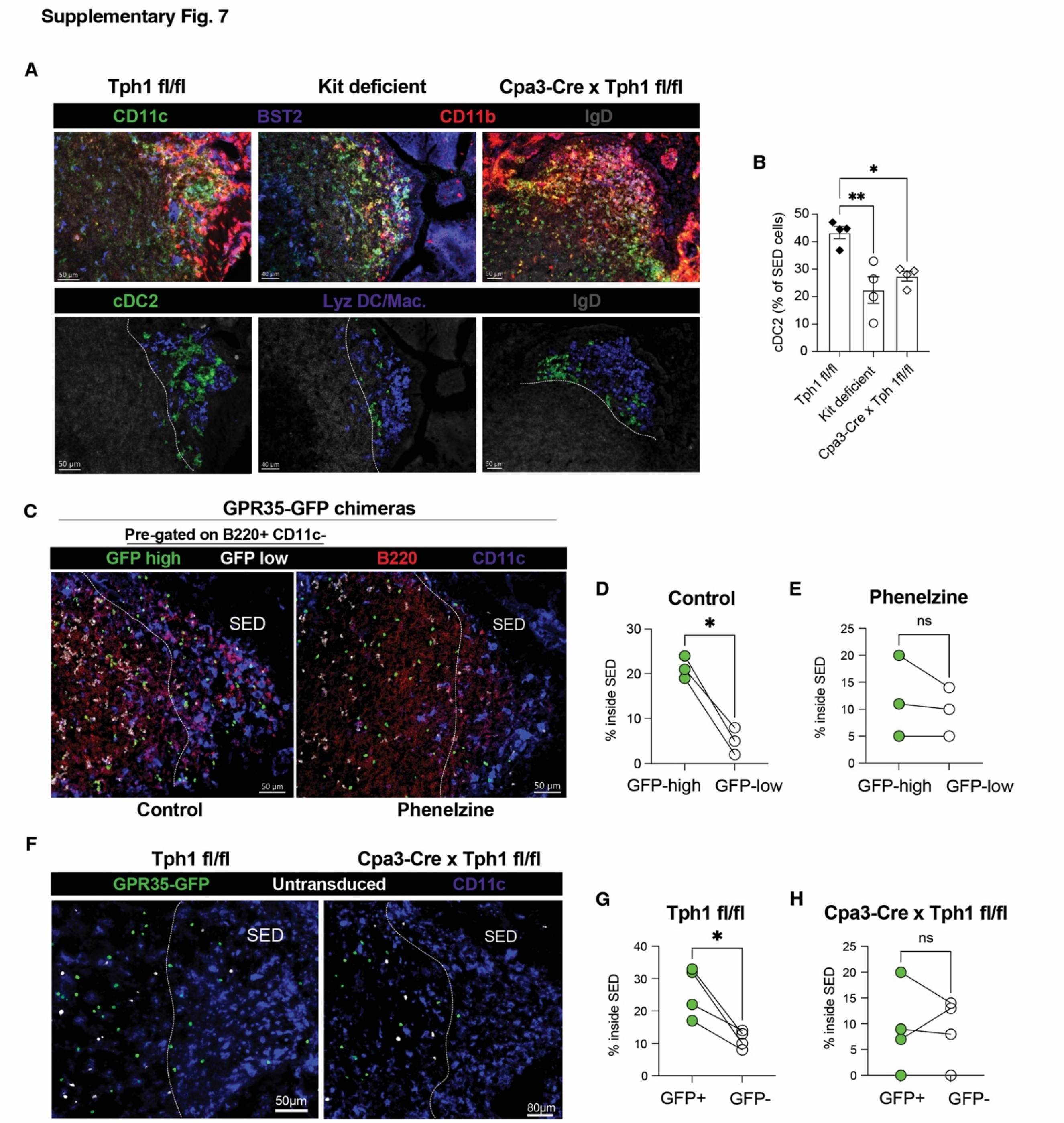

Supplementary Figure 7. Mast cell-derived serotonin metabolite(s) support cell positioning within SED. A. Immunofluorescence micrographs showing PPs from Tph1$^{fl/fl}$ (left), mast cell deficient Kit/v x Kit/W (middle) and Cpa3-Cre Tph1$^{fl/fl}$ (right) mice. CD11c (green), BST2 (blue), CD11b (red) and IgD (grey) staining (top), or cDC2 (green, bottom) and

Lyz DC/Mac (blue, bottom) are shown. SED= subepithelial dome. White dashed lines identify the SED and were depicted based on BST2 and IgD staining. **B**. Quantification of cDC2 % in the SED of Tph1$^{fl/fl}$, mast cell deficient and Cpa3-Cre x Tph1$^{fl/fl}$ mice from images shown in **A**. *n*=4. Data are pooled from 2 independent experiments. **C**. Immunofluorescence micrographs showing PPs from control (left) or phenelzine treated (right) GPR35-GFP overexpressing chimeras. GFP high (green), GFP low (white), CD11c (purple) staining are shown. **D, E**. Quantification of GPR35-GFP high and low cell % in SED of control (**D**) or phenelzine treated (**E**) GPR35-GFP overexpressing chimeras, from images of the type shown in **C**. *n*=3. Data are pooled from 2 independent experiments. **F**. Immunofluorescence micrographs showing PPs from Tph1$^{fl/fl}$ (left) and Cpa3-Cre x Tph1$^{fl/fl}$ (right) that received Deep Red-labeled GPR35-GFP overexpressing cells, 60 hrs after transfer. Deep Red$^+$ GFP$^+$ (green), Deep Red$^+$ GFP$^-$ (untransduced, white) and CD11c (blue) staining are shown. **G, H**. Quantification of GPR35-GFP$^+$ and GFP$^-$ % (out of total CD11C$^+$ CD11b$^+$ cells) in SED of Tph1$^{fl/fl}$ (**G**) and Cpa3-Cre Tph1$^{fl/fl}$ (**H**) mice from images of the type shown in **F**. *n*=4. Data are pooled from 2 independent experiments. * $p<0.05$; ** $p<0.005$; *** $p<0.0005$. Data are presented as mean ± SEM. Paired- t-tests (**D-H**) and ordinary one-way ANOVA using Turkey's multiple comparisons test (**B**) were performed.

Supplementary Fig. 8

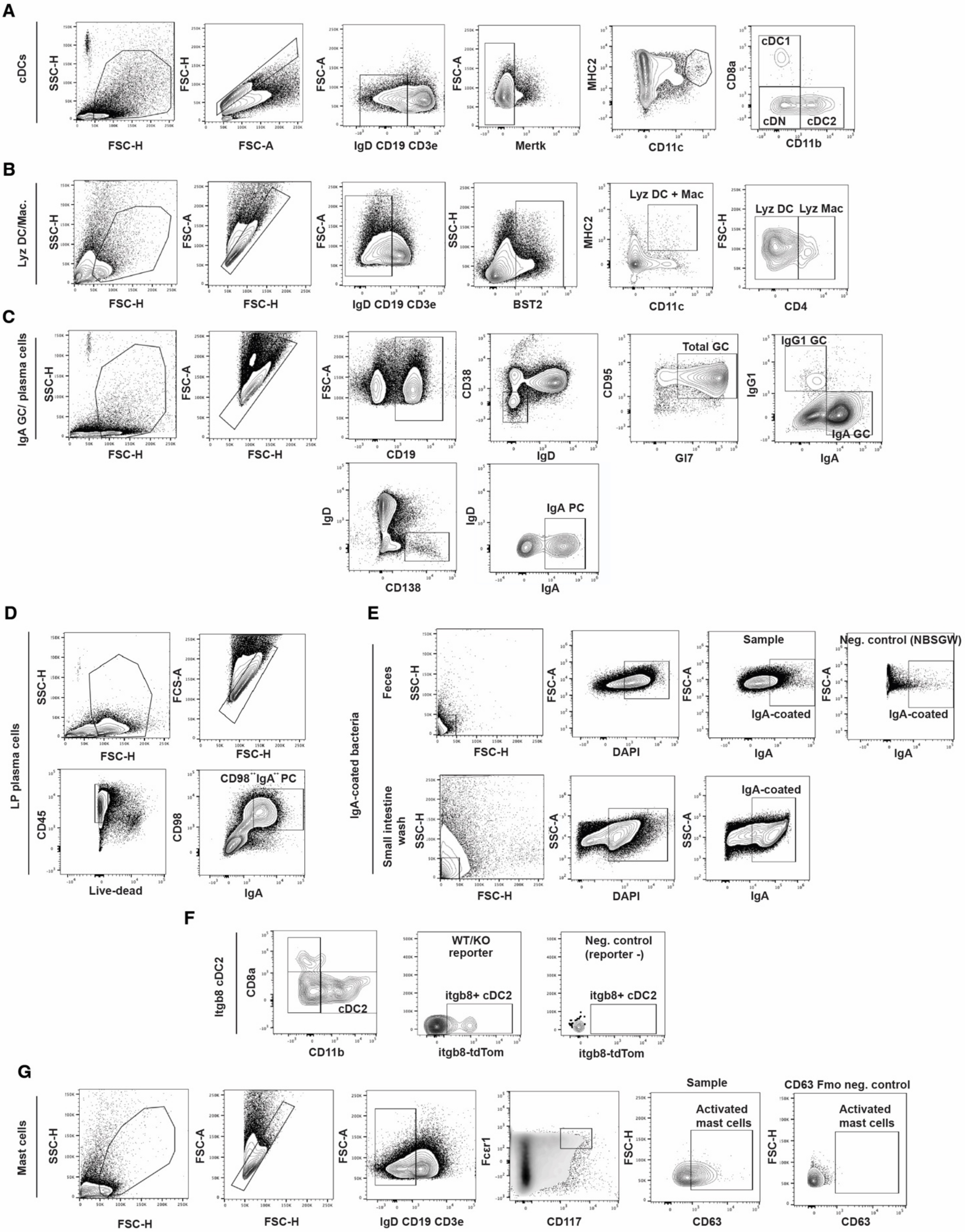

Supplementary Figure 8. Flow cytometry gating strategies.

A-G. Representative examples of cDC (**A**), Lyz DC and Lyz MAC (**B**), IgA GC and plasma cell (**C**), lamina propria plasma cells (**D**), IgA-coated intestinal bacteria (**E**), itgb8-expressing cDC2 (**F**), and CD63$^+$ mast cell (**G**) gating strategies are shown.

Supplementary Movie Legends

Supplementary movie 1.

3D reconstruction of whole-mount SED imaging showing CD45.1 (red), CD45.2 (blue), CD11c (white) and BST2 (green) staining (left); or cDC WT (red), cDC KO (blue), BST2 (green) gated populations (right), from CD45.1 WT/ CD45.2 GPR35$^{+/-}$ control mixed chimeras.

Supplementary movie 2.

3D reconstruction of whole-mount SED imaging showing CD45.1 (red), CD45.2 (blue), CD11c (white) and BST2 (green) staining (left); or cDC WT (red), cDC KO (blue), BST2 (green) gated populations (right), from CD45.1 WT/ CD45.2 GPR35$^{-/-}$ mixed chimeras.
